# Multi-Year Mortality Due to Staphylococcal Arthritis and Osteomyelitis with Sandspur-Associated Injury in Juvenile Black Skimmers (*Rynchops niger*) at Nesting Colonies in Southwest Florida, USA

**DOI:** 10.3390/vetsci11110578

**Published:** 2024-11-18

**Authors:** Nicole M. Nemeth, Janell M. Brush, W. Andrew Cox, Rebecca Hardman, Brittany Piersma, Alexandra Troiano, Heather W. Barron, Melanie R. Kunkel, Chloe C. Goodwin, Alisia A. W. Weyna, Amy S. McKinney, Xuan Hui Teo, Rebecca Radisic, Lisa A. Shender, Susan Sanchez, Michelle van Deventer

**Affiliations:** 1 Southeastern Cooperative Wildlife Disease Study, University of Georgia, Athens, GA 30602, USA; mrk266@cornell.edu (M.R.K.); chloecgoodwin@uga.edu (C.C.G.); aweyna@wisc.edu (A.A.W.W.); xuan.teo@uga.edu (X.H.T.); rradisic@ucdavis.edu (R.R.); 2 Department of Pathology, University of Georgia, Athens, GA 30602, USA; 3 Florida Fish and Wildlife Conservation Commission, Fish and Wildlife Research Institute, Gainesville, FL 32611, USA; janell.brush@myfwc.com; 4 Florida Fish and Wildlife Conservation Commission, Gainesville, FL 32601, USA; william_cox@fws.gov; 5 Florida Fish and Wildlife Conservation Commission, Fish and Wildlife Research Institute, St. Petersburg, FL 33701, USA; rebecca.hardman@myfwc.com (R.H.); lisa_shender@nps.gov (L.A.S.); 6 Florida Fish and Wildlife Conservation Commission, Naples, FL 32601, USA; brittany@audubonwe.org (B.P.); m.vandeventer@tnc.org (M.v.D.); 7 Clinic for the Rehabilitation of Wildlife, Sanibel, FL 33957, USAhbarron@marinelife.org (H.W.B.); 8 Athens Veterinary Diagnostic Laboratory, University of Georgia, Athens, GA 30602, USA; amymc@uga.edu (A.S.M.); ssanchez@uga.edu (S.S.)

**Keywords:** seabird, colonial nest, shorebird, disease, bacterial arthritis, sandspur, microbial resistance, polyarthritis, *Staphylococcus*

## Abstract

The black skimmer (*Rynchops niger*) is a state-threatened, colonially nesting seabird in Florida, USA. Conservation threats include habitat alteration, human disturbances, severe weather, and predation. During nest monitoring (May–September, 2020–2022), black skimmer juveniles at colonies on Fort Myers Beach and Marco Island, Florida, had polyarthritis and died or were euthanized due to severe illness. Similarly-aged skimmers from geographically distant (considered unaffected) colonies were evaluated for comparison (2021–2023). We documented field, clinical, radiographical, and pathological findings to characterize disease and purported pathogenesis. The majority were lame and lethargic, in poor nutritional condition, and dehydrated. Additionally, 8/23 of the skimmers with dermatitis and arthritis from affected colonies also had penetrating sandspurs associated with skin ulceration, scabbing, and/or hemorrhage. The affected joints were often in limbs (interphalangeal and hock; less commonly stifle, elbow, carpus). A postmortem evaluation and bacteriology revealed *Staphylococcal aureus*-associated dermatitis, arthritis, tenosynovitis, and/or osteomyelitis in 21/22 of the juvenile skimmers from southwestern nest colonies. *Staphylococcus aureus* dissemination to internal organs occurred in 10/13 of the skimmers tested. Among skimmers evaluated from distant colonies, 5/10 that were examined histologically had skin crusting and inflammation but lacked arthritis. Occasional coinfections were documented (e.g., West Nile virus, Gram-negative bacilli). The results suggest that staphylococcal joint disease originated from sandspur-induced skin damage, followed by hematogenous dissemination to the joints and, occasionally, the internal organs. Additional nest sites should be tested to evaluate disease risk and potentially contributing environmental factors. We recommend that site managers employ techniques that reduce the risk of skimmer interactions with sandspurs.

## Simple Summary

Black skimmers are state-threatened, colonial nesting seabirds that face numerous conservation health challenges. Through regular nest colony surveys, we observed a concerning pattern of annual fatalities among black skimmer juveniles that had grossly swollen joints at several nest colonies. This joint disease affected their mobility and ability to thrive and led to severe wasting and death in some individuals. Clinical and postmortem examinations of skimmers for four sequential years revealed that the joints were infected with a bacterium, *Staphylococcus aureus*, which normally cohabits the skin of many species (including humans) without causing disease. However, in this case, *S. aureus* likely gained entry to joints via skin injuries from sandspurs, which arise from vegetation that is common to many Florida beaches. *S. aureus* is also commonly detected as a sand and water contaminant in popular recreational beaches, which may also serve as a source of exposure in the skimmers. We recommend continued monitoring of black skimmer nest colonies for arthritic disease and other health-related challenges, with consideration of management techniques to reduce the risk of sandspur–skimmer interactions at nesting sites.

## 1. Introduction

The order Charadriiformes, which is composed of a diverse array of species (e.g., sandpipers, plovers, avocets, oystercatchers, skimmers, and others), is undergoing population declines in the face of global conservation challenges, such as habitat degradation and loss, anthropogenic activities, pollution and contaminants, and adverse weather events [1,2]. Alarming rates of decline across many species groups in North America include a 21.5% decrease in seabird abundance from 1970 to 2017, with 51.9% of seabird species in decline [3]. The black skimmer (*Rynchops niger*) is a colonially nesting, migratory seabird whose geographic range includes portions of eastern and western North, Central, and South America, with utilization of both coastal and riverine habitats [2]. In Florida, a U.S. state with a steadily increasing human population (currently at >22 million) [4], the black skimmer is a state-threatened species, with nesting colonies along the coast that range from a few to several hundred pairs [1,5].

In addition to the considerable underlying population pressures on seabirds and other coastal-dependent birds, infectious agents, such as viruses and bacteria, are an ever-looming threat. This threat became starkly evident when A/goose/Guangdong/1/1996 lineage highly pathogenic (HP) clade 2.3.4.4b H5N1 influenza A virus (IAV) was introduced to North America in late 2021 [6]. This virus has since led to fatal outcomes in many native American seabird species, among them the black skimmer, dunlin (*Calidris alpina*), sanderling (*Calidris alba*), snowy plover (*Anarhynchus nivosus*), and ruddy turnstone (*Arenaria interpres*) [7]. However, a paucity of information exists on other infectious agents that may threaten seabirds, specifically the black skimmer. The proximity and overlap of important black skimmer habitat (e.g., beaches) in areas of heavy human use, as well as exposure to potentially contaminated waters, elevates threats of infection with zoonotic pathogens, such as *Staphylococcus* and *Salmonella* spp. [8]. Additionally, environmental stressors, such as poor-quality habitat and increasingly frequent or intense weather events, may further diminish immunologic defenses, thereby increasing susceptibility to infections in many wildlife species. We characterized a multi-year bacterial disease outbreak among juvenile black skimmers on nesting beach colonies in southwestern Florida, USA.

During sequential nesting seasons (May–September 2020, 2021, and 2022), black skimmer nestlings and fledglings at nest colonies on beaches in southwest Florida were often observed with multiple severely swollen joints in the legs and wings. Further, many of the birds were entangled with sandspurs. Sandspurs are thorny seed capsules produced by a number of sandbur species, including southern sandbur (*Cenchrus echinatus* L.) and coastal sandbur (*C. spinifex*), which are native grasses that germinate in late spring to late summer/early fall [9], which correlates with black skimmer nesting season. In response to this concerning, multi-year, seasonal outbreak, our overall aim was to characterize the disease and its cause in the context of potential ongoing population health risks to inform mitigation strategies and, thus, support conservation efforts for this state-threatened species. The specific achieved objectives included (1) characterizing clinical observations of black skimmers in the field and clinic; (2) assessing radiographic and pathologic changes in affected skimmers, both in the joints and the whole body; (3) determining the etiologic agent of joint disease; (4) assessing for coinfections and comorbidities; (5) collectively evaluating field, clinical, pathological, and microbiological data to postulate pathogenesis and gauge potential ongoing health risks; and (6) informing mitigation strategies to improve nesting success at black skimmer nesting colonies in southwest Florida.

## 2. Materials and Methods

### 2.1. Field and Clinical Observations

Routine, annual monitoring of black skimmer nest colonies in Florida occurs through the coordinated efforts of the Florida Shorebird Alliance (FSA), which provides annual monitoring data, survey reports, and action plans [10]. Observations of skimmer chicks with reduced mobility (lameness), lethargy, and general poor health were reported at Fort Myers Beach and Marco Island in Lee and Collier Counties, Florida, respectively, during the nesting season (May–September) from 2020 to 2022. These Gulf beaches are considered affected and are hereafter referred to as “southwestern [nest] colonies”. Two distant nesting colonies (Lido Beach, Sarasota County, and Redington Shores, Pinellas County) approximately 150 miles to the north of the southwestern sites were also monitored during the breeding season as part of the FSA network, were evidently unaffected (i.e., negative control), and are hereafter referred to as “distant [nest] colonies”. These four nesting colonies are relatively similar in size (>100 breeding pairs) and proximity to human recreation and development. Nesting areas above the high tide line are closed to public access, with symbolic fencing (i.e., notifications at edge boundaries to warn people to keep their distance) at the beginning of each nesting season, but the wet sand and lower beach remain open to pedestrian access and recreational use. Beach morphology (slope, width) varies among sites, as does dune vegetation composition and density. Varying degrees of vegetation management occur at each of the sites outside of the nesting season but are generally suspended during the nesting season while eggs and flightless chicks are present. Ongoing monitoring efforts in the field included the routine handling of flightless skimmers (including chicks) for identification (i.e., marking) and physical examination. The latter revealed swollen joints in some mostly juvenile individuals. Some of those were found dead or died within a day of handling on the beach. Others were admitted to wildlife rehabilitation clinics but either died during transport or were euthanized via a parenterally administered euthanasia solution (pentobarbital sodium) within 24 h due to poor prognosis. Clinical assessments were made by wildlife biologists in the field, as well as by veterinarians and veterinary staff at wildlife rehabilitation clinics. In-clinic diagnostic evaluations of skimmers included in this study consisted of radiographs of five individuals and a joint tap in one individual. All skimmer handling and care adhered to standards consistent with state and federal wildlife rehabilitation permits.

### 2.2. Postmortem Evaluation

Following natural death or euthanasia due to severe morbidity, the carcasses of 35 skimmers (33 juveniles and 2 adults) were refrigerated and either necropsied at the Florida Fish and Wildlife Conservation Commission (FWC) facilities or shipped overnight to the Southeastern Cooperative Wildlife Disease Study (SCWDS) at the University of Georgia, College of Veterinary Medicine, where they underwent full postmortem evaluation. When necropsies were conducted at the FWC, fresh tissues and formalin-fixed samples were collected and sent to the SCWDS for further evaluation. The nutritional condition was determined as poor, fair, or good based on the evaluation of adipose in subcutaneous tissues, overlying muscle over the throat (furcula bone), pectoral and abdominal muscles over the heart, and overlying internal viscera, as well as the robustness of pectoral and leg musculature and a histologic evaluation of bone marrow, when available. The animal was deemed emaciated when a histologic bone marrow evaluation revealed serous atrophy of fat (multiple bones examined); fat was grossly absent; and musculature was atrophied. Because not all skimmers underwent a histologic bone marrow evaluation, emaciation and poor nutritional condition were categorized together.

Tissue samples representative of major organ systems (i.e., heart, trachea, lung, liver, kidney, spleen, skeletal muscle (pectoral), esophagus, proventriculus, ventriculus, small and large intestines, pancreas, joints, including bone and overlying skin and skeletal muscle, brain, gonads, and cloacal bursa (when grossly visualized)) were collected at necropsy and preserved in 10% neutral-buffered formalin. In addition, fresh samples of kidney, liver, lung, brain, heart, and joint tissue and swabs were frozen at −20 °C. Formalin-fixed tissues were routinely sectioned at a 4 um thickness for histopathology and stained with hematoxylin and eosin and Brown and Brenn Gram stain at the Histology Laboratory in the Department of Pathology, University of Georgia, an American Association of Veterinary Laboratory Diagnosticians (AAVLD)-accredited laboratory.

### 2.3. Aerobic Bacterial Culture and Sensitivity Testing

Ancillary tests were performed when indicated by postmortem findings and for the surveillance of potential pathogens in some cases. Joint tissue and/or swabs from 18 skimmers with grossly swollen joints from southwestern (i.e., affected) nesting colonies and 1 skimmer with swollen joints from a distant colony underwent aerobic bacterial culture at the Athens Veterinary Diagnostic Laboratory, University of Georgia, an AAVLD-accredited laboratory. In addition, samples from select organs were tested based on histopathology findings (i.e., discrete bacterial colonies within the parenchyma), as well as numerous sandspurs that were aseptically collected directly from chicks or beaches from which the affected skimmers originated, at the time that the birds were discovered as sick or dead. Sandspurs collected from sites and timing corresponding to skimmer cases W21-517 and W21-481 were aseptically handled and swabbed, and swabs were cultured. In some cases, samples underwent one to two freeze–thaw cycles prior to bacterial culture.

All culture samples were plated on trypticase soy agar with 5% sheep blood (BD 221261), MacConkey agar (Thermo Scientific R01552 (Waltham, MA, USA)), and Columbia CNA agar with 5% sheep blood (BD 221353). Respiratory samples were also plated on chocolate agar (Thermo Scientific R01300). Plates were incubated for 72 h at 42 °C in ambient air. Respiratory samples were incubated at 42 °C in CO_2_ conditions. Plates were examined each day for growth. Colonies were identified using Vitek Maldi-TOF (bioMérieux, Salt Lake City, UT, USA). Susceptibilities were performed using the Vitek^®^ 2 (bioMérieux) instrument with Gram-positive antimicrobial susceptibility testing (AST) cards AST-GP81 (bioMérieux) for the following antimicrobials: amikacin, amoxicillin/clavulanic acid, benzylpenicillin, cefalotin, cefovecin, cefoxitin screen, cefpodoxime, chloramphenicol, clindamycin, doxycycline, enrofloxacin, erythromycin, florfenicol, gentamicin, inducible clindamycin resistance, marbofloxacin, minocycline, nitrofurantoin, oxacillin, pradofloxacin, and trimethoprim/sulfamethoxazole. The AST-GP81 card was automatically inoculated with a bacterial suspension generated in a 0.45% sodium chloride solution at spectrophotometric turbidity of 0.5. Inoculum preparation and card filling generally were separated by less than 30 min and put into the instrument for incubation and reading. The Vitek^®^ 2 Advanced Expert SystemTM was used to provide categorical interpretations of the Vitek^®^ 2 AST data. Identification was performed on the three most predominant organisms. In addition, seven *S. aureus* isolates (two from skimmers affected in 2020; three from skimmers affected in 2021; and two from skimmers affected in 2022) representing both joint and lung/kidney samples underwent antimicrobial sensitivity testing using Vitek^®^ 2 g-positive susceptibility card (AST-GP78; bioMérieux), which included the following panel of antimicrobials: benzylpenicillin, cefoxitin screen, ceftaroline, ciprofloxacin, clindamycin, daptomycin, doxycycline, gentamicin, inducible clindamycin resistance, moxifloxacin, nitrofurantoin, oxacillin, quinupristin/dalfopristin, rifampicin, tetracycline, tigecycline, trimethoprim/sulfamethoxazole, and vancomycin. Interpretation was based on minimum inhibitory concentration (MIC) values (µg/mL), and criteria were based on human and animal data available via the Clinical and Laboratory Standards Institute and drug manufacturers. MIC ranges according to antibiotics, bacterial species, animal (host) species, and the anatomic location of infection/sample type are provided by the manufacturer.

### 2.4. Additional Laboratory Testing

Additional diagnostic testing on select cases was performed to assess for concurrent infections. Virus isolation aimed at detection of West Nile virus and eastern equine encephalitis virus was performed on pooled tissues (heart, kidney, brain); virus isolates were subjected to a West Nile virus reverse transcription PCR test [11]. A PCR test for avian influenza virus on pooled oropharyngeal and cloacal swabs [12], avian paramyxoviruses on cloacal swabs [13], with modifications in extraction methods as described [14], herpesvirus at AVDL, and *Mycoplasmopsis* spp. on joint swabs or tissue was conducted via real-time PCR methods, as described [15], with adaption to a conventional PCR. These tests were performed in select cases primarily aimed at the detection of subclinical or pre-existing infections (lesions suggestive of these recognized potential pathogens were not observed).

Brevetoxin enzyme-linked immunosorbent assay (ELISA; PbTx-3 eq) testing and microcystin/nodularin testing were performed on select cases at the Harmful Algal Blooms Laboratory, Ecosystem Assessment and Restoration Section at the Fish and Wildlife Research Institute, Florida Fish and Wildlife Conservation Commission (FWC). The brevetoxin detection threshold was 5–10 ng/g (wet weight), and the tissues tested variably included kidney, liver, ventriculus contents, and intestinal contents. The kidney and liver were tested for microcystins and nodularins using the 2-methyl-3-methoxy-4-phenylbutyric acid (MMPB) method with a detection threshold of 5 ng/g (wet weight).

## 3. Results

### 3.1. Demographic, Geographic, and Clinical Observations

**Figure 1:**
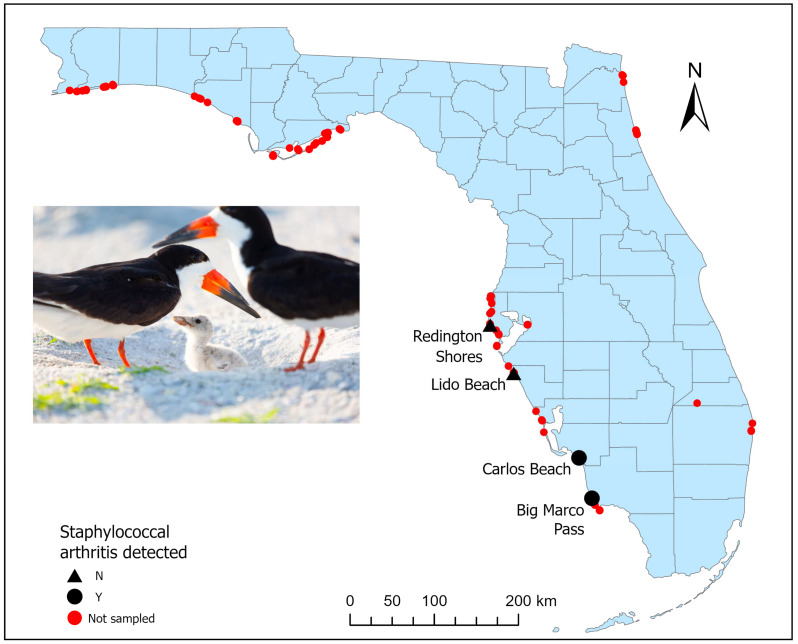
Map of Florida, USA, indicating nesting areas in which staphylococcal arthritis was detected in black skimmers (*Rynchops niger*) from 2020 to 2022, as well as areas in which evaluated skimmers did not exhibit this disease (2021–2023).

A total of 35 severely moribund or dead black skimmers were evaluated during nest seasons (May to September) from 2020 to 2023 (Table 1). Twenty-three of these (twenty-two juveniles, one adult; twelve males, two females, and nine of unknown sex) were located in nesting colonies along coastal areas of southwestern Florida counties (Lee and Collier) that are known to have skimmers affected by staphylococcal arthritis, while twelve skimmers (eleven juveniles, one adult; two males, four females and six of unknown sex) were from more central-western counties (Sarasota and Pinellas; Figure 1) that are not known to have skimmers affected by staphylococcal arthritis. The birds collected from southwestern colonies likely represented a subset of the number of juvenile skimmers affected. Due to high summer temperatures and scavenging by ants and ghost crabs, most skimmer carcasses were deemed unsuitable for necropsy. However, the FWC estimated that in 2020, 50% of hatch-year birds died at Florida’s largest skimmer colony within the Big Marco Pass Critical Wildlife Area (CWA). In 2021, ~35% of hatch-year birds died at Big Marco Pass CWA, and ~20% died at a second colony on Carlos Beach in Ft. Myers [16].

**Figure 2:**
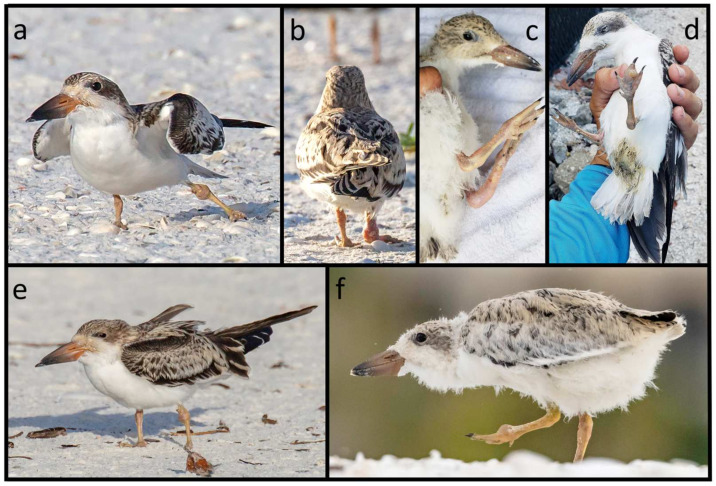
Juvenile black skimmers that exhibited markedly swollen tibiotarsal–metatarsal (hock; (**a**,**c**,**e**)) and interphalangeal (digit; (**b**,**d**,**f**)) joints in Florida, USA, during annual nest monitoring. Photographs for a, b, e, and f are courtesy of Jean Hall.

**Figure 3:**
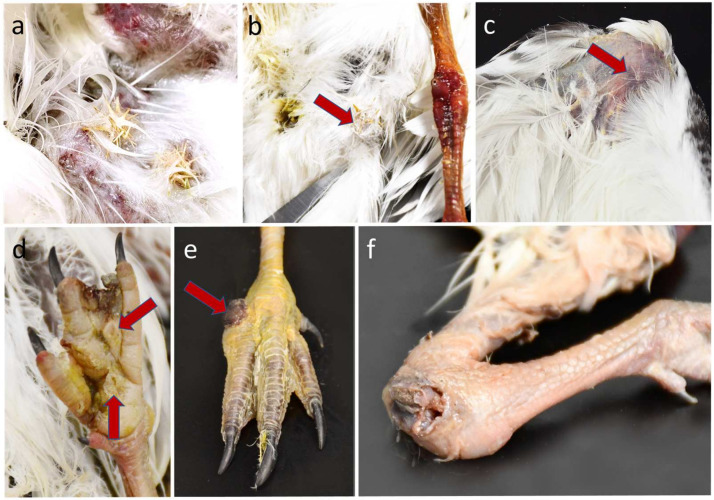
Areas of attached sandspurs in feathers and surrounding skin of the inguinal region (**a**) and near the tail base, adjacent to hock joint (red arrow) (**b**). Presumed skin puncture wounds (red arrow) in the skin over the wing (carpus, (**c**)) and plantar aspect of foot pad and digits (**d**). Severe skin ulceration (red arrows, **d**) and/or scabbing (red arrow) in the proximal digit (**e**) and hock joints (**f**) of juvenile black skimmers in Florida, USA.

For those observed alive, clinical signs included poor nutritional condition, dehydration, and weakness; mentation varied from alert to dull and lethargic, and some were unable to stand. Their joints were variably swollen, including combinations of femorotibiotarsal (stifle), tibiotarsal-tarsometatarsal (hock), tarsometatarsal–phalangeal, interphalangeal (Figure 2), and less commonly, humeroradioulnar (elbow) and ulnar–radial–carpometacarpal (carpus). Anecdotally, sandspurs penetrated the nonfeathered skin of the feet, legs, and/or wings and sometimes were enmeshed in the body feathers of skimmers observed in the field and in the clinic. These findings were corroborated by full or partial (i.e., broken off) sandspurs penetrating skin in 8/23 of those that underwent postmortem evaluation (Table 1). An additional nine skimmers (17/23 total) had findings consistent with penetrating skin wounds of varying chronicity, sometimes over the joints (wings, legs and/or digits; Figure 3), and associated with inflammation, ulceration, and hemorrhage in some cases. Radiographic findings in five skimmers with clinical arthritis were consistent with marked osteoarthritis (Figure S1), occasionally with osteomyelitis (bony lysis) and pathologic bone fractures. A microscopic examination of a joint tap from one skimmer at a rehabilitation clinic revealed Gram-positive cocci.

### 3.2. Pathology

Grossly, all 35 evaluated skimmers across all sites (23 from southwestern beaches, 12 from more northern beaches) were in poor to emaciated nutritional condition, with scant to absent adipose stores and atrophic skeletal musculature (Figure S2), as well as serous atrophy of fat in those for which bone marrow was histologically examined (Table S1). Most had scant gastrointestinal contents (sometimes with few fish bones and sand); two had one or more macroplastic fragments in the ventricular lumen. Birds examined in the field had variable (often moderate to high) burdens of feather lice and mites (ectoparasites were often lost from carcasses during storage and transport). Many had pericloacal fecal and/or urate staining. Other salient gross lesions in skimmers with arthritis were rare and included focal mild hepatic pallor (microscopically corresponding to a granuloma) in one skimmer and multifocal mild, yellow, minimally raised nodules on the renal surface (microscopically corresponding to necroheterophilic nephritis) in another. Pulmonary edema was observed in two other skimmers, and, rarely, subjective splenomegaly and/or hepatomegaly was observed.

Overall, endoparasite loads were low and considered non-contributory to illness. Parasites in juvenile skimmers with staphylococcal polyarthritis included small numbers of fluke adults and/or eggs in the renal collecting ducts of two skimmers; few intestinal cestodes in one skimmer; and few intestinal nematodes in another. At distant (nonaffected) nest colonies, one skimmer had few trematodes in the renal collecting ducts similar to those in arthritic skimmers; another had small numbers of esophageal trematodes; and two others had intestinal luminal trematodes (Table S1).

**Figure 4:**
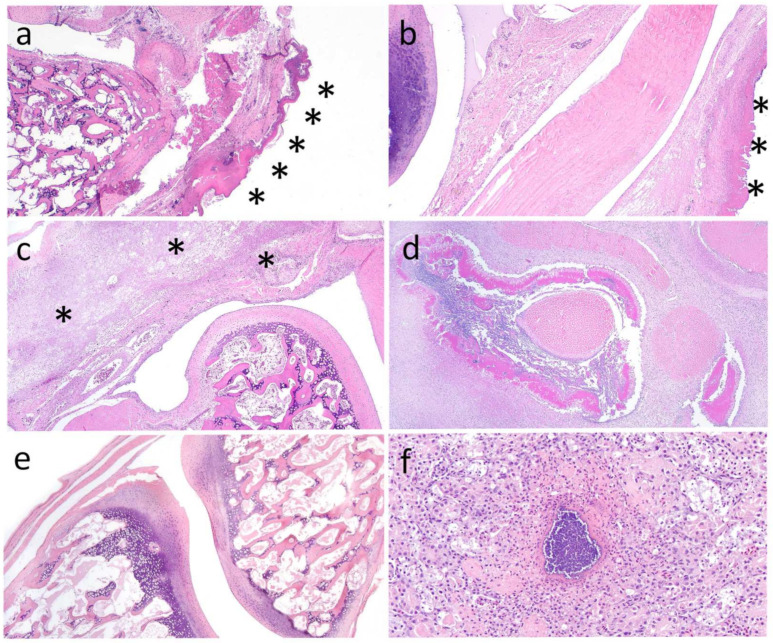
(**a**) Skin wound over the hock joint of a non-*Staphylococcus aureus*-infected juvenile black skimmer (ID: W23-482B); the epidermis is necrotic and hyperkeratotic (asterisks), with underlying lymphoplasmacytic dermatitis and extension of inflammation to associated joint (magnification 2×). (**b**) Skin wound over the hock joint of a *S. aureus*-infected, juvenile black skimmer (ID: W22-520A), with epidermal ulceration, necroheterophilic histiocytic dermatitis, and superficial coccoid bacteria, along with heterophilic, histiocytic synovitis (magnification 2×). (**c**) The periarticular space of the hock joint (surrounding the head of the tibiotarsus) exhibits necrosis and is markedly expanded by edema and heterophilic, histiocytic, and lymphoplasmacytic inflammation extending to the adjacent tendon and overlying dermis of a skimmer with *S. aureus* infection (W21-481D; magnification 4×). (**d**) Necroheterophilic inflammation with embedded bacterial cocci surrounding a tendon near the tibiotarsus of a black skimmer with *S. aureus* infection (W21-481D); the surrounding joint space is expanded by heterophilic, histiocytic, and lymphoplasmacytic inflammation (magnification 4×). (**e**) Normal hock joint for comparison; note the clear (white) joint space (W21-673F (magnification 4×)). (**f**) Kidney with a dense colony of *S. aureus* bacteria surrounded by a rim of heterophils with pyknosis and fibrin in a black skimmer (W21-481C) with disseminated *S. aureus* infection (magnification 20×). (**a**–**f**) Hematoxylin and eosin stain.

The majority (21/22; 95.5%) of black skimmer juveniles from southwestern colonies and 0/11 juveniles (12 total, with a single adult) from distant colonies had grossly swollen joints. For skimmers in southwestern colonies, most often, multiple joints were affected, commonly involving interphalangeal (digits) and tibiotarsal–metatarsal (hock) joints (Figure S3) and, less commonly, femoral–tibiotarsal joints (stifle), ulnar–radial–carpometacarpal (carpus) joints, and humeroulnar and radial (elbow) joints. One skimmer from a southwestern colony and one from a distant colony were adults; neither had evidence of arthritis, dermatitis, superficial skin lesions, or sandspurs; these are excluded from the denominators hereafter. Three skimmers with hindlimb arthritis had bone fractures of the tibiotarsus (2) and femur (1), all of which were in the distal diaphysis (i.e., adjacent to the joint). Scabs and crusting on the skin and abrasions or puncture wounds on the plantar foot surfaces and/or over leg joints and/or featherless areas of wings, sometimes with hemorrhages, were noted in many skimmers with arthritis (16/22; 72.7% at southwestern colonies; 4/11 [36.4%] at northern colonies; Table S1). These were often associated with sandspurs or broken-off sandspur spikes embedded in the skin, including interdigital webbing (Table 1). Histologically, affected juvenile skimmers in southwestern colonies had epidermal, and sometimes dermal, ulceration with associated heterophilic, granulomatous, and/or lymphoplasmacytic infiltration with necrosis. Severe inflammation, necrosis, and Gram-positive bacterial cocci extended deep to surround, and sometimes infiltrate and invade, adjacent tendons and/or bones in grossly affected joints; these regions were sometimes associated with Gram-positive, large coccoid bacteria. In ten individuals with staphylococcal arthritis, Gram-positive bacteria morphologically consistent with *Staphylococcus* spp. disseminated to distant organs (i.e., kidney and/or lung; Figure 4, Table 1; Figure 4e depicts skin overlying a joint and bone absent of lesions, for comparison). Unlike in skimmers from southwestern colonies, histopathology in skimmers from distant colonies did not include arthritis, tenosynovitis, or osteomyelitis. However, among the ten examined histologically, five (50%) had skin lesions, including ulceration, crusting, and heterophilic dermatitis (gross skin lesions were in 6/11; Table S1).

Apart from skin and joint lesions, histopathology in skimmers with staphylococcal arthritis included three with disseminated staphylococcal infections involving heterophilic inflammation, necrosis, and Gram-positive cocci in the lung, heart, and/or kidney. Two others had disseminated Gram-negative infections that included heterophilic and granulomatous inflammation, with occasional Gram-negative bacteria in the liver, lung, kidney, intestine, and/or bone marrow. Two skimmers from the same colony had focally extensive, moderate, necrotizing pancreatitis with fibrosis (with no evident cause). Two others had renal lesions suggestive of dehydration (urate stasis, tubular luminal mineralized concretions with mild tubular damage); three had incidental parasitism (two with renal trematodes and one with intestinal cestodes). The single adult from the southwestern colonies was diagnosed with spindle cell hepatocellular carcinoma based on neoplastic cell morphology, and the single adult from the distant colonies was diagnosed with emaciation and hepatitis (suspected due to Gram-negative bacilli; Table 1 and Table S1).

### 3.3. Bacteriology and Antimicrobial Sensitivity Testing

Heavy to moderate growth of *S. aureus* was cultured from affected joint samples from all 18 skimmers from southwestern colonies from which it was attempted (Table 1). Among these, a heavy (and usually pure) growth of *S. aureus* was also isolated from the lung or kidney, which were selected for culture based on histopathology (see above). Additional rare isolates from these internal organ samples also included *Enterococcus faecalis*. A culture of the liver from one skimmer from a southwestern site that had arthritis yielded light to moderate growth of *Escherichia coli*, *Pseudomonas aeruginosa*, and *Enterococcus faecalis*. A culture of swabs of sandspurs collected from southwestern colonies with affected juvenile skimmers yielded no growth from six samples from Fort Myers Beach (case W21-517), while two swabs collected from Marco Island (case W21-481) yielded light growth of *Enterococcus faecalis*, *Mammaliicoccus* (formerly *Staphylococcus*) *sciuri*, and *Pantoea dispersa* and heavy growth of *E. faecalis*, *Staphylococcus xylosus*, and *Acinetobacter radioresistens*.

A bacterial culture of the only skimmer from a distant colony for which a joint sample was assessed (without corresponding inflammation) yielded light growth of *M. sciuri* and *S. xylosus*. Joint tissue from another skimmer with chronic dermatitis on the skin over the joints of the legs and digits and the neck was cultured, resulting in the growth of a single *M. sciuri* colony.

A bacterial culture of the internal organs (lung, kidney, and/or liver) from ten skimmers with *S. aureus* polyarthritis at southwestern sites isolated *S. aureus*. Growth was heavy to moderate in 7/10 and light in 2; however, a culture was performed after ≥3 freeze–thaw cycles in the latter two cases. Tissues from which *S. aureus* was cultured included lungs from seven skimmers and kidneys from three skimmers (both tissues were tested in two birds). The culture also resulted in the heavy growth of *E. faecalis* from the lungs of four skimmers and the kidney of one skimmer, from which *S. aureus* was cultured. From the skimmer from which no *S. aureus* was cultured, only the liver was tested and yielded light to moderate growth of *Escherichia coli*, *Pseudomonas aeruginosa*, and *Enterococcus faecalis*. In this case, the liver was cultured because Gram-negative bacilli were evident histologically within a heterophilic granuloma (Table 1). In one case, despite histologic visualization of intralesional Gram-negative bacterial bacilli, a culture was not performed due to highly pathogenic avian influenza (HPAI) virus-based biosafety protocols (the case was suspected HPAI virus-positive based on an initial PCR result, which was later determined negative through confirmatory testing at the National Veterinary Services Laboratory, Ames, IA [12]).

A bacterial culture of internal organs (lung and/or liver) from six skimmers without pathologic evidence of *S. aureus* arthritis, all from distant colonies, yielded moderate growth of *S. aureus* from the lung of one skimmer. This sample also yielded moderate growth of *M. sciuri* and light growth of *Rothia mucilaginosa*. Lungs from two other skimmers from distant colonies yielded light growth of *E. faecium* from one and moderate growth of *E. faecalis* and light of *M. sciuri* and *Escherichia coli* from another lung. There was no growth from the livers or lungs of the remaining three skimmers.

Antimicrobial sensitivity testing on isolates from seven skimmers yielded mostly consistent results, indicating susceptibility (S) to all antimicrobials tested, with intermediate susceptibility (I) only demonstrated in one skimmer (W21-673A), which was to cefpodoxime (third generation; Table S2).

### 3.4. Other Ancillary Test Results 

West Nile virus was isolated from the tissues of 2/13 of the skimmers tested from southwestern sites and 0/8 from distant colonies. There was no evidence of avian influenza viruses in 26 skimmers (15 from affected, 11 from nonaffected sites) or paramyxoviruses in 12 skimmers (10 affected colonies, 2 nonaffected) using PCR test or pan (consensus) herpesvirus PCR test in 2 (1 from affected, 1 from non) skimmers. *Mycoplasmopsis* spp. DNA was not detected in seven skimmers tested from affected sites and one from a distant colony.

No *Mergibacter septicus* (formerly Bisgaard taxon 40) was detected using an aerobic bacterial culture [17,18] of joint samples from 20 skimmers (including 18 from southwestern colonies and 2 from distant colonies) or in internal organ samples from 18 skimmers (including 12 from southwestern colonies and 6 from distant colonies).

Detectable brevetoxin concentrations were in tissues and/or gastrointestinal contents of 2/15 of the skimmers tested from southwestern colonies and 4/7 of the skimmers from distant colonies. Microcystins/nodularins were not detected in tissues from two skimmers from distant colonies (Table 1).

## 4. Discussion

We attributed black skimmer annual mortality events to staphylococcal arthritis. Juvenile black skimmers were diagnosed with fatal staphylococcal arthritis and emaciation for three sequential breeding seasons at nesting colonies in southwestern Florida. An imperiled species in Florida, the black skimmer relies on sandy coastal habitats for nesting [1,2] that often are shared with human beachgoers. Infectious disease is uncommonly reported in black skimmers and seabirds in general, while recognized conservation challenges most commonly include habitat alteration, adverse and extreme weather events, predation risk, and anthropogenic activities [1,2,5,19]. Prey base and provisioning are additional important considerations; however, based on our comprehensive diagnostic evaluation of these black skimmers, we consider that emaciation was secondary to staphylococcal joint disease. Skimmers have diverse foraging tactics and move around fresh, brackish, and saltwater areas, making them more likely to be able to adapt to shifts in the abundance of any single species or foraging location [20]. The black skimmer breeding population in North America experienced a significant decline in population size between 2012 and 2022 [21], although a clearer understanding of this trend is hindered by high variation in annual estimated population numbers in some colonies [2]. Concurrent with improving our understanding of population dynamics, recognizing health threats to local and regional populations is critical, as they may have broader and potentially long-term implications for black skimmer conservation and statewide recovery efforts.

Staphylococcal arthritis and aspects of pathogenesis have been described in poultry [22] (pp. 995–1003). For *S. aureus* to successfully infect a host, host defense mechanisms, such as skin or mucous membrane barriers, must be compromised. Once this barrier is breached, the bacteria may access the joint via direct extension (depending on the anatomic location of entry and depth of wound) or gain access to the circulation and can disseminate to joints, while inciting inflammation and tissue damage in the surrounding bone, tendons, and muscle. Clinical manifestation likely depends on the entry point and dose. Affected birds may be febrile, lame, and lethargic and remain recumbent, with drooped wings; the disease may culminate in fatal starvation and dehydration. Field, clinical, and pathology findings in juvenile black skimmers suggest a similar pathogenesis of *S. aureus* arthritis, with possible progression to osteomyelitis, as in poultry [22] (pp. 995–1003).

In black skimmers, our findings suggest that *S. aureus* entry and infection were facilitated by sandspur-induced skin injuries over the feet, legs, wings, and ventrum, allowing bacterial entry locally that spread to nearby joints, followed, in some cases, by hematogenous dissemination to other areas of the body. Gross and histopathology in some skimmers corroborated this suspicion and included acute to subacute (i.e., partially healed) open (puncture) skin wounds, including ulceration with variable scabbing, crusting, sloughing, and surrounding inflammation, with the extension of bacteria deep into the underlying dermis and other soft tissues (muscle, tendons, bone). In some cases, hemorrhage was evident on the skin and feathers of the wings, legs, and body adjacent to puncture wounds and/or embedded sandspurs. Blood loss from repeated sandspur-induced injuries could further contribute to weakness and lethargy. As can occur in poultry, *S. aureus* in some skimmers disseminated to parenchymal organs (e.g., kidney and lung). Warmer temperatures may facilitate the development of septicemia in poultry [22] (pp. 995–1003), which is a trend that also may occur in black skimmers, as the nesting season takes place during the Florida summer (i.e., the hottest time of the year) [2,23]. Death may occur rapidly after infection, but with a more prolonged disease course, joints exhibit chronic inflammation with infiltration of tendons, synovial membranes, and adjacent soft tissues [22] (pp. 995–1003). All affected skimmers were emaciated or in poor nutritional condition, and some had evidence of dehydration, which was likely secondary to bacterial joint infections. Painful joints and skin wounds could deter an inexperienced, juvenile bird from feeding and possibly alienate it from parental care, prompting a rapid decline in health. The young age and corresponding underdeveloped immune systems likely rendered them more prone to opportunistic bacterial infection and may have further facilitated disease development and severity. Rarely, skimmers had evidence of other infections and/or diseases (e.g., Gram-negative bacilli, such as *Escherichia coli* and West Nile virus), which also suggests weakened health status and a predilection to secondary infections.

*S. aureus* colonizes the skin, nares, and oropharynx of humans and many other animals, including livestock, companion animals, and wildlife, all of which are considered potential reservoirs [24,25]. All avian species are susceptible to infection, and the bacterium is commonly isolated from healthy wild animals [22] (pp. 995–1003), [24]. Further, an estimated 30% of asymptomatic people carry *S. aureus* in their nasal cavities [26]. Thus, the bidirectional transfer of *S. aureus* and other potential pathogens across human, domestic animal, and wildlife interfaces is an important epidemiologic consideration, especially for individuals with underdeveloped (i.e., young) and compromised immune systems, which are most vulnerable to severe disease development [27]. *S. aureus*, along with numerous enteric pathogens, represents an ongoing and costly public health threat, with over USD 900 million expended annually on the human health impacts of marine-borne pathogens in the U.S. [28]. Further, the development of antimicrobial resistance and high virulence strains, including methicillin-resistant *S. aureus* (often referred to as MRSA), represent a monumental global challenge to both human and veterinary health [22] (pp. 995–1003) [24,26]. Fortunately, *S. aureus* isolates from skimmers in our study did not suggest the development of antimicrobial resistance.

In some situations, humans may serve as a source of *S. aureus*, as well as other bacteria, to wildlife populations [24]. Transmission among humans often occurs via direct skin-to-skin contact [26], but *S. aureus* also may be shed in feces [24] and on sloughed skin [27]. In addition to skin, the bacterium can remain viable in the environment, including on substrates and in the air and water [23] (pp. 995–1003) [24,25]. *S. aureus* is relatively resistant to adverse conditions, including high salinity, pH extremes, dryness, and high temperatures [25]. For example, increased levels of viable *S. aureus* have been detected in sand and marine and freshwater beaches frequented by higher densities of people [25,27,29,30]. In some areas, *S. aureus* prevalence and or levels were higher in beaches in close proximity to wastewater treatment plants [30] and during warmer, wetter months [27], as occurs in Florida when black skimmer nesting coincides with the hot and rainy summer months [23]. The ever-increasing cohabitation of coastal areas by people, domestic animals, and wildlife, the latter of which may be forced into suboptimal or fragmented habitats, could collectively contribute to an increased risk of infection and disease among all groups [24]. In addition to *S. aureus*, other bacteria could pose a threat at high-density human beaches [27,29], as suggested by our study findings that several skimmers had evidence of infection (and in one case, associated disease) with Gram-negative bacterial bacilli, including *Enterococcus* sp. and *Escherichia coli*. Further, black skimmers at another Florida nesting colony had been previously diagnosed with salmonellosis associated with sewage overflow [8].

In addition to juvenile skimmers with staphylococcal arthritis, sandspur-associated skin injuries, and malnutrition in nest colonies in southwestern Florida, juvenile skimmers at distant Florida nest colonies suffered from skin injuries and inflammation. Some of the latter had puncture wounds or abrasions either directly associated with or suspected to be due to penetrating sandspur-induced injuries. The majority of those examined microscopically had associated skin lesions, including the skin overlying joints. Despite the lack of *S. aureus* joint infections documented in the population of skimmers at distant colonies, the skin wounds likely contributed to pain, stress, and poor nutritional status. Evidence, albeit less common, of sandspur-associated skin damage, emaciation, and death in juvenile skimmers at these more distant colonies suggests that *S. aureus* may only be a potential contributing, but not a necessary, factor in juvenile skimmer mortalities. Rather, sandspurs and associated skin wounds may pose the greater threat, and the density and distribution of sandspurs at these sites (i.e., potential for contact) are important future considerations. Juvenile skimmers are likely more prone to sandspur contact as they seek shade and refuge in dune vegetation during the hottest, busiest times of the day at the beach, which is a behavior often observed at colonies by nest monitors in our study.

While pre-existing infections (e.g., viral) can facilitate the development of secondary bacterial infections [22] (pp. 995–1003), we rarely documented evidence of coinfections in skimmers with staphylococcal arthritis. West Nile virus was detected in two skimmers, but these likely represented either very early or subclinical infections because lesions consistent with West Nile virus were not evident [31]. Other bacteria, in addition to heavy growth of *S. aureus*, were isolated from joint samples and were often presumed contaminants when growth was light; however, in several cases, Gram-negative bacilli were associated with lesions and disease. Two years of our study (2022–2023) overlapped with a severe HP IAV outbreak that affected a high number and diversity of wild bird species [7,32]. While no skimmers in our study tested positive for HP IAV, as of September 2024, the USDA reported two black skimmers that tested positive for HP IAV with associated morbidity/mortality; one was in Sarasota County, Florida, and the other was in Nassau County, New York. Both occurred from September to November of 2022 [7]. Despite these few detections in skimmers, seabirds in general remain at risk of HP IAV-associated death, potentially in high numbers [7,33,34]. In addition to infectious agents, toxins and other contaminants may weaken the immune system and lead to other physiologic changes that may predispose to severe infections [35]. Heavy metal assessment, including mercury and lead, would have provided useful data in the skimmers, but the amount of available tissue samples (i.e., in smaller birds) needed for testing was a limiting factor in toxicologic testing. The significance of plastics in the mid-gastrointestinal tract is unknown in the present cases but is of high concern in general in aquatic birds, in which it can cause extensive gastrointestinal tract scarring that could affect survival [36]. Finally, there was no gross or histologic evidence of metabolic bone disease [37].

Low levels of brevetoxins were detected in tissues and/or gastrointestinal contents of a few black skimmers in our study, but this included a higher proportion of those from the distant colonies, so it is not suspected to have played a role in staphylococcal disease development or morbidity. However, consideration of the role of brevetoxins and other environmental toxins in seabird health is critical. Southwest Florida is affected by harmful algal blooms of *Karenia brevis* or “Florida red tides” annually, and because these black skimmer chicks presented with hind limb weakness and ataxia, as previously reported in wild birds with brevetoxicosis [38], this was considered a differential diagnosis. Even after septic arthritis was identified, algal toxins remained a potential concern because sepsis is a common sequela in many birds with brevetoxicosis, and some environmental toxins diminish immunocompetence [35].

## 5. Conclusions

The extent of the ongoing risks posed by biological and mechanical hazards to seabirds that utilize beach habitats is unknown, but our study reveals a concerning mortality pattern in juvenile black skimmers in some nesting colonies. Black skimmers and other aquatic and piscivorous birds can serve as sentinels for the health of aquatic environments, and some of these species also face an indefinite future [2]. Other potential contributors to disease development that can be difficult to account for include suboptimal environmental conditions (e.g., habitat or quality or availability of prey) at breeding sites; these are also a continued concern for skimmer and other seabird populations [2]. Additional investigations at black skimmer nest colony sites in Florida are ongoing to better characterize the microbiome of individual skimmers and the presence of *S. aureus* in nearshore waters and beach sands. Genetic sequencing of *S. aureus* isolates has also been initiated to compare with human and environmental isolates and across skimmer colonies. These results will be reported separately. To improve black skimmer nestling survival, we recommend that site managers employ management techniques that reduce the risk of sandspur interaction with chicks and fledglings during the nesting season and continue to monitor for staphylococcal arthritis and other diseases in both juvenile and adult skimmers. Further, considering regional challenges in the broader context of climatic and environmental changes that may contribute to long-term stress among seabird populations is imperative.

## Figures and Tables

**Table 1:** Location and date of morbidity and/or mortality, demographic information, and diagnostic data on general lesion patterns, bacteriology, and brevetoxin exposure in black skimmers from nest colonies along the western Florida coast from 2020 to 2023.

ID	Location, County	Date Died/Euthanized	Age	Sex	General Gross/ Histologic Lesion Pattern ^1−3^	Skin Wounds or Sandspurs ^5^	*S. aureus* Cultured from Joint	*S. aureus* Cultured from Viscera	Brevetoxins (ng/g) (Tissue[s] Tested) ^6^
**Colonies affected by staphylococcal arthritis (southwest coast)**									
W20-456A	Marco Island, Collier	23 August 2020	Juvenile	Female	A, D	S	Yes	No (liver)	U (L)
W20-456B	Marco Island, Collier	23 August 2020	Juvenile	Male	A, D	S	Yes	No (liver)	U (L)
W20-456C	Marco Island, Collier	23 August 2020	Juvenile	Male	A, D	P	NT	NT	U (L)
W20-456D	Marco Island, Collier	23 August 2020	Juvenile	Unknown	A, D	S	NT	NT	NT
W20-456E	Marco Island, Collier	23 August 2020	Juvenile	Unknown	A, D	S, P	NT	NT	NT
W21-481A ^3^	Marco Island, Collier	20 July 2021	Juvenile	Male	A, D	SS	Yes	NT	U (L, K)
W21-481B ^3^	Marco Island, Collier	20 July 2021	Juvenile	Male	A, D	SS	Yes	NT	U (L, K)
W21-481C	Marco Island, Collier	22 July 2021	Juvenile	Male	A, DB	None	Yes	Yes (kidney)	U (L, K)
W21-481D	Marco Island, Collier	22 July 2021	Juvenile	Male	A, D	P	Yes	NT	U (L, K)
W21-481E ^3,7^	Marco Island, Collier	22 July 2021	Adult	Male	N	None	NT	NT	NT
W21-517B ^3,7^	Little Estero Island, Fort Myers Beach, Lee	22 July 2021	Juvenile	Male	A, D	SS, S, P	Yes	No (lung)	U (L, K)
W21-517C ^3,7^	Little Estero Island, Fort Myers, Lee	22 July 2021	Juvenile	Male	A, D	SS, S, P	Yes	Yes (lung)	U (L, K, V)
W21-673A ^3,7,9^	Fort Myers, Lee	21 September 2021	Juvenile	Unknown	A, D, DB	SS, S, P	Yes	Yes (lung)	19.9 (K)
W21-673B ^3,7,9^	Marco Island, Collier	21 September 2021	Juvenile	Unknown	A, D, DB	P	Yes	Yes (lung)	NT
W21-673D ^3,7,9^	Marco Island, Collier	21 September 2021	Juvenile	Male	A, D	SS, P	Yes	Yes (lung)	NT
W21-673E ^3,7,9^	Marco Island, Collier	21 September 2021	Juvenile	Unknown	A, D ^4^	None	Yes	NT	U (K)
W21-673G ^3,7,9^	Fort Myers, Lee	21 September 2021	Juvenile	Unknown	A, D	SS, S	Yes	Yes (lung)	U (K)
W21-673H ^3,7,9^	Fort Myers, Lee	21 September 2021	Juvenile	Male	A, D, DB ^4^	SS, S	Yes	Yes (lung)	NT
W21-673I ^3,7,9^	Unknown	21 September 2021	Juvenile	Unknown	A, D	S	Yes	Yes (lung)	NT
W22-520A	Carlos Beach, Lee	13 July 2022	Juvenile	Female	A, D, DB	U	Yes	Yes (kidney)	12 (IC); U (L, K, VC)
W22-520B	Carlos Beach, Lee	13 July 2022	Juvenile	Unknown	A, D	U	Yes	NT	U (L, K, IC, VC)
W22-520C	Carlos Beach, Lee	13 July 2022	Juvenile	Unknown	A, D, DB	P	Yes	Yes (kidney); No (lung)	U (L, K, IC, VC)
W22-647	Big Marco Island, Collier	23 August 2022	Juvenile	Male	DB	None	NT	NT	NT
**Distant colonies unaffected by staphylococcal arthritis (northwest coast)**									
W21-673C ^3^	Lido Key Beach, Sarasota	21 September 2021	Juvenile	Unknown	N	None	NT	Yes (lung)	41.4 (K)
W21-673F	Lido Key Beach, Sarasota	21 September 2021	Juvenile	Unknown	D	None	No	NT	22.5 (K)
W22-410A	Lido Beach, Sarasota	7 June 2022	Adult	Female	N	None	NT	No (liver)	U (L, K, IC) ^8^
W22-410B	Lido Beach, Sarasota	7 June 2022	Juvenile	Unknown	N	None	NT	NT	NT
W22-486	Lido Beach, Sarasota	1 July 2022	Juvenile	Female	N	None	NT	NT	NT
W22-645 ^3^	Redington Shores, Pinellas	23 August 2022	Juvenile	Unknown	N	P	NT	NT	NT
W23-482A	Lido Key Beach, Sarasota	12 July 2023	Juvenile	Female	D	SS, P	NT	No (lung)	U (L, V, I)
W23-482B	Lido Key Beach, Sarasota	12 July 2023	Juvenile	Male	D	P	No	No (liver)	U (L, V, I)
W23-482C	Lido Key Beach, Sarasota	12 July 2023	Juvenile	Male	D	P	NT	No (lung, cloacal bursa)	8.2 (I); U (L, V)
W23-482D	Lido Key Beach, Sarasota	12 July 2023	Juvenile	Female	D	SS, S, P	NT	No (lung)	14.2 (L); U (V, I)
W23-482E	Lido Key Beach, Sarasota	12 July 2023	Juvenile	Unknown	N	None	NT	NT	NT
W23-482F	Lido Key Beach, Sarasota	12 July 2023	Juvenile	Unknown	N	S, P	NT	NT	NT

^1^ All skimmers were in poor to emaciated nutritional condition, and many had evidence of dehydration. ^2^ A—arthritis; D—dermatitis and/or dermal necrosis; N—neither; DB—disseminated bacterial disease. ^3^ Necropsy performed at Florida Fish and Wildlife Conservation Commission facilities; all others necropsied at the Southeastern Cooperative Wildlife Disease Study. ^4^ West Nile virus was isolated from tissues with no characteristic histopathology of West Nile disease. ^5^ S—skin scabbing and/or hemorrhage; P—skin puncture wounds, small lacerations or ulcerations; SS—sandspurs or spurs embedded in skin. ^6^ U—undetectable (<5 ng/g); NT—not tested; L—liver; K—kidney; V—ventriculus; I—intestine; C—contents. ^7^ No *Mycoplasmopsis* spp. were detected in joint samples using PCR test. ^8^ No microcystins or nodularins detected (liver, kidney; <5 ng/g). ^9^ No paramyxoviruses were detected in cloacal swabs using PCR test.

## Data Availability

The original contributions presented in this study are included in the article/Supplementary Material; further inquiries can be directed to the corresponding author/s.

## Supplementary Materials

The following supporting information can be downloaded at https://www.mdpi.com/article/10.3390/vetsci11110578/s1: Figure S1: Radiographs revealing marked swelling of soft tissue region surrounding the tibiotarsal–tarsometatarsal (hock) joint in a black skimmer with staphylococcal arthritis; Figure S2: Poor nutritional condition with absent adipose stores and markedly atrophic skeletal musculature in a black skimmer with staphylococcal arthritis; Figure S3: Black skimmers with staphylococcal arthritis suspected to be associated with sandspurs often exhibited skin ulceration and necrosis (evident as darkening and sloughing) (a,b), marked swelling of one or more joints (b–e) with pale, yellow, soft to firm material (inflammation; (c,e)). Joint disease most often involved interphalangeal (digits, (a–c)) and tibiotarsal–metatarsal (hock, (b–e)) joints; Table S1: Clinical, gross, and histopathologic findings and final diagnosis of black skimmers from southwestern and geographically distant nesting colonies along the western coast of Florida from 2020 to 2023; Table S2: Antimicrobial susceptibility pattern for *Staphylococcus aureus* isolates from juvenile black skimmers with staphylococcal polyarthritis in from nest colonies along southwest Florida from 2020 to 2023.

## References

[1] Florida Fish and Wildlife Conservation Commission (FWC) (2013). A Species Action Plan for Four Imperiled Species of Beach-Nesting Birds.

[2] Gochfeld M., Burger J., Lefevre K.L., Billerman S.M. (2020). Black Skimmer (*Rynchops niger*), Version 1.0. Birds of the World.

[3] Rosenberg K.V., Dokter A.M., Blancher P.J., Sauer J.R., Smith A.C., Smith P.A., Stanton J.C., Panjabi A., Helft L., Parr M. (2019). Decline of the North American avifauna. Science.

[4] Macrotrends, “Florida Population 1900–2023”. https://www.macrotrends.net/states/florida/population#:~:text=The%20population%20of%20Florida%20in,a%201.9%25%20increase%20from%202021.

[5] Florida Fish and Wildlife Conservation Commission (FWC) (2011). Biological Status Review Report for the Black Skimmer (Rynchops niger).

[6] Caliendo V., Lewis N.S., Pohlmann A., Baillie S.R., Banyard A.C., Beer M., Brown I.H., Fouchier R.A.M., Hansen R.D.E., Lameris T.K. (2022). Transatlantic spread of highly pathogenic avian influenza H5N1 by wild birds from Europe to North America in 2021. Sci. Rep..

[7] U.S. Department of Agriculture, Animal and Plant Health Inspection Service 2024 Detections of Highly Pathogenic Avian Influenza in Wild Birds. https://www.aphis.usda.gov/aphis/ourfocus/animalhealth/animal-disease-information/avian/avian-influenza/hpai-2022/2022-hpai-wild-birds.

[8] Shender L.A., Cody T., Ruder M., Fenton H., Niedringhaus K.D., Blanton J., Motes J., Schmedes S., Forys E. (2022). Heavy rainfall, sewer overflows, and salmonellosis in black skimmers (*Rynchops niger*). Ecohealth.

[9] Sellers B., Smith H., Ferrell J. Document SS-AGR-364. Identification and Control of Southern Sandbur (*Cenchrus echinatus* L.) in Hayfields. University of Florida, Institute of Food and Agricultural Sciences. 2018 (Revised). https://edis.ifas.ufl.edu/publication/AG373.

[10] Florida Shorebird Alliance. https://flshorebirdalliance.org/.

[11] Allison A.B., Mead D.G., Gibbs S.E., Hoffman D.M., Stallknecht D.E. (2004). West Nile virus viremia in wild rock pigeons. Emerg. Infect. Dis..

[12] Youk S., Torchetti M.K., Lantz K., Lenoch J.B., Killian M.L., Leyson C., Bevins S.N., Dilione K., Ip H.S., Stallknecht D.E. (2023). H5N1 highly pathogenic avian influenza clade 2.3.4.4b in wild and domestic birds: Introductions into the United States and reassortments, December 2021–April 2022. Virology.

[13] Kim L.M., Suarez D.L., Afonso C.L. (2008). Detection of a broad range of class I and II Newcastle disease viruses using a multiplex real-time reverse transcription polymerase chain reaction assay. J. Vet. Diagn. Investig..

[14] Das A., Spackman E., Pantin-Jackwood M.J., Suarez D.L. (2009). Removal of real-time reverse transcription polymerase chain reaction (RT-PCR) inhibitors associated with cloacal swab samples and tissues for improved diagnosis of avian influenza virus by RT-PCR. J. Vet. Diagn. Investig..

[15] Rebelo A.R., Parker L., Cai H.Y. (2011). Use of high-resolution melting curve analysis to identify *Mycoplasma* species commonly isolated from ruminant, avian, and canine samples. J. Vet. Diagn. Investig..

[16] Van Deventer M., Brush J.M., Cox A., Hardman R. (2024). Personal communication.

[17] De Luca E., Álvarez-Narváez S., Maboni G., Baptista R.P., Nemeth N.M., Niedringhaus K.D., Ladner J.T., Lorch J.M., Koroleva G., Lovett S. (2021). Comparative genomics analyses support the reclassification of Bisgaard taxon 40 as *Mergibacter* gen. nov., with *Mergibacter septicus* sp. nov. as type species: Novel insights into the phylogeny and virulence factors of a Pasteurellaceae family member associated with mortality events in seabirds. Front. Microbiol..

[18] Niedringhaus K.D., Shender L.A., DiNuovo A., Flewelling L.J., Maboni G., Sanchez S., Deitschel P.J., Fitzgerald J., Nemeth N.M. (2021). Mortality in common (*Sterna hirundo*) and sandwich (*Thalasseus sandvicensis*) terns associated with Bisgaard Taxon 40 infection on Marco Island, Florida, USA. J. Comp. Pathol..

[19] Fink D., Auer T., Johnston A., Strimas-Mackey M., Ligocki S., Robinson O., Hochachka W., Jaromczyk L., Crowley C., Dunham K. (2023). eBird Status and Trends, Data Version: 2022.

[20] Forys E.A., Hevesh A.R. (2017). Investigating Black Skimmer Chick Diets Using Citizen Science and Digital Photography. Southeast. Nat..

[21] Paleczny M., Hammill E., Karpouzi V., Pauly D. (2015). Population trend of the world’s monitored seabirds, 1950–2010. PLoS ONE.

[22] Andreasen C.B., Swayne D.E. (2020). Staphylococcosis. Diseases of Poultry.

[23] National Oceanic and Atmospheric Administration (NOAA), “ASOS Climate Normals”. https://www.weather.gov/tbw/newnormals_asos.

[24] Heaton C.J., Gerbig G.R., Sensius L.D., Patel V., Smith T.C. (2020). *Staphylococcus aureus* epidemiology in wildlife: A systematic review. Antibiotics.

[25] Topić N., Cenov A., Jozić S., Glad M., Mance D., Lušić D., Kapetanović D., Mance D., Vukić Lušić D. (2021). *Staphylococcus aureus*—An additional parameter of bathing water quality for crowded urban beaches. Int. J. Environ. Res. Public Health.

[26] Chambers H.F., Deleo F.R. (2009). Waves of resistance: *Staphylococcus aureus* in the antibiotic era. Nat. Rev. Microbiol..

[27] Esiobu N., Green M., Echeverry A., Bonilla T.D., Stinson C.M., Hartz A., Rogerson A., McCorquodale D.S. (2013). High numbers of *Staphylococcus aureus* at three bathing beaches in South Florida. Int. J. Environ. Health Res..

[28] Ralston E.P., Kite-Powell H., Beet A. (2011). An estimate of the cost of acute health effects form food- and water-born marine pathogens and toxins in the United States. J. Water Health.

[29] Goodwin K.D., McNay M., Cao Y., Ebentier D., Madison M., Griffith J.F. (2012). A multi-beach study of *Staphylococcus aureus*, MRSA, and enterococci in seawater and beach sand. Water Res..

[30] Thapaliya D., Hellwig E.J., Kadariya J., Grenier D., Jefferson A.J., Dalman M., Kennedy K., DiPerna M., Orihill A., Taha M. (2017). Prevalence and characterization of *Staphylococcus aureus* and methicillin-resistant *Staphylococcus aureus* on public recreational beaches in northeast Ohio. Geohealth.

[31] Steele K.E., Linn M.J., Schoepp R.J., Komar N., Geisbert T.W., Manduca R.M., Calle P.P., Raphael B.L., Clippinger T.L., Larsen T. (2000). Pathology of fatal West Nile virus infections in native and exotic birds during the 1999 outbreak in New York City, New York. Vet. Pathol..

[32] Nemeth N.M., Ruder M.G., Poulson R.L., Sargent R., Breeding S., Evans M.N., Zimmerman J., Hardman R., Cunningham M., Gibbs S. (2023). Bald eagle mortality and nest failure due to clade 2.3.4.4 highly pathogenic H5N1 influenza a virus. Sci. Rep..

[33] Hall J.S., Franson J.C., Gill R.E., Meteyer C.U., TeSlaa J.L., Nashold S., Dusek R.J., Ip H.S. (2011). Experimental challenge and pathology of highly pathogenic avian influenza virus H5N1 in dunlin (*Calidris alpina*), an intercontinental migrant shorebird species. Influenza Other Respir. Viruses.

[34] Pardo-Roa C., Nelson M.I., Ariyama N., Aguayo C., Almonacid L.I., Munoz G., Navarro C., Avila C., Ulloa M., Reyes R. (2023). Cross-species transmission and PB2 mammalian adaptations of highly pathogenic avian influenza A/H5N1 viruses in Chile. bioRxiv.

[35] Acevedo-Whitehouse K., Duffus A.L. (2009). Effects of environmental change on wildlife health. Philos. Trans. R. Soc. Lond. B Biol. Sci..

[36] Charlton-Howard H.S., Bond A.L., Rivers-Auty J., Lavers J.L. (2023). ‘Plasticosis’: Characterizing macro- and microplastic-associated fibrosis in seabird tissues. J. Hazard. Mater..

[37] Klasing K.C., Korver D.R., Swayne D.E. (2020). Nutritional Diseases. Diseases of Poultry.

[38] Fauquier D.A., Flewelling L.J., Maucher J.M., Keller M., Kinsel M.J., Johnson C.K., Henry M., Gannon J.G., Ramsdell J.S., Landsberg J.H. (2013). Brevetoxicosis in seabirds naturally exposed to *Karenia brevis* blooms along the central west coast of Florida. J. Wildl. Dis..

