# Supplementary material for: Multi-Year Mortality Due to Staphylococcal Arthritis and Osteomyelitis with Sandspur-Associated Injury in Juvenile Black Skimmers (Rynchops niger) at Nesting Colonies in Southwest Florida, USA
Source: Vet Sci. 2024 Nov 18;11(11):578. doi: 10.3390/vetsci11110578 (PMC11598991; doi:10.3390/vetsci11110578)
Supplement: Supplementary file 1 [file vetsci-11-00578-s001.zip › vetsci-3251662-supplementary.pdf]

Supplemental Table 1. Clinical, gross and histopathologic findings and final diagnosis of black skimmers from southwestern and geographically distant nesting colonies along the western coast of Florida from 2020-2023.

ID	Reported clinical/field history and disposition	Gross pathology[1,2]				Histopathology			Final diagnoses
		Weight (g)	Joints affected[3] and lesion traits	Skin	Other	Joints (select joints evaluated)	Skin overlying affected joints	Other[4]	
**Colonies affected by staphylococcal arthritis (southwest coast)**									
**W20-456A**	Lameness<br>Lethargy<br>Swollen joints (legs/digits and wings)<br>Radiographs: Osteomyelitis, left carpus and right digits; severe osteomyelitis with pathologic fracture, right hock<br>Euthanized in clinic	140.0	Hock and stifle: severe bilateral swelling	Skin scabs, bilateral and multifocal over medial tibiotarsi and digits	Fracture closed, distal right tibiotarsus<br>Focal hepatic pallor	Left hock, tarsometatarsophalanges, digits: Multifocal, severe, subacute, heterophilic, lymphoplasmacytic tenosynovitis and osteomyelitis with hyperkeratosis, edema and gram-negative bacilli and gram-positive cocci	Multifocal, epidermal ulceration with serocellular crusts, orthokeratotic hyperkeratosis with gram-positive cocci and gram-negative, short bacilli; multifocal, moderate heterophilic, lymphoplasmacytic dermatitis with edema, necrosis and myriad, deep gram-negative short bacilli and gram-positive cocci	Focal, mild, subacute, hepatic, heterophilic granuloma with gram negative, short bacilli<br>Multifocal, mild, subacute, heterophilic and plasmacytic pneumonia	Staphylococcal dermatitis, arthritis, tenosynovitis and osteomyelitis with skin ulceration and crusting<br>Hepatic granuloma (*Escherichia coli*, *Pseudomonas aeruginosa* isolated)<br>Poor nutritional condition
**W20-456B**	Lameness<br>Lethargy<br>Swollen joints (legs/digits and wings)<br>Radiographs: Osteomyelitis with pathologic fracture and severe bony changes, distal left tibiotarsus and proximal tarsometatarsus; osteomyelitis, right digits 3 and 4<br>Euthanized in clinic	170.0	Hock: severe bilateral swelling<br>Digits: moderate unilateral (right) swelling, digits 3 and 4<br>Tarsometatarsal-phalangeal joint: moderate bilateral swelling<br>Hip: moderate unilateral (left) joint laxity	Skin scabs multifocally over left foot	Fracture, left tibiotarsus	Left hock, tarsometatarsophalanges, digits: Multifocal, severe, subacute, heterophilic, lymphoplasmacytic tenosynovitis and osteomyelitis with hyperkeratosis, edema and gram-negative bacilli and gram-positive cocci	Multifocal, mild epidermal ulceration with serocellular crusts, orthokeratotic hyperkeratosis and gram-positive cocci and gram-negative, short bacilli; multifocal, marked heterophilic, lymphoplasmacytic dermatitis with edema and necrosis	None	Staphylococcal dermatitis, arthritis, tenosynovitis and osteomyelitis with skin ulceration and crusting<br>Hepatic granuloma (Escherichia coli, Pseudomonas aeruginosa isolated)<br>Poor nutritional condition

**W20-456C**	Lameness<br>Lethargy<br>Swollen joint (legs/digits, wings)<br>Radiographs: Osteomyelitis, left elbow, right and left carpi, left hock, right metatarsophalangeal joint, left digit 3; large osteophyte, left tibiotarsus mid-diaphysis<br>Euthanized in clinic	210.0	Carpus and elbow: unilateral (left) rigidity<br>Hock: mild unilateral (left) swelling and nodule<br>Digits: mild bilateral plantar swelling, digit 3; unilateral (right) nodule digit 4	Superficial skin abrasion, left carpus	None	Left hock, tarsometatarsophalanges, digit: Multifocal, moderate, subacute to chronic, heterophilic, lymphoplasmacytic tenosynovitis and osteomyelitis with hyperkeratosis, edema and bacilli and cocci	Multifocal, epidermal ulceration with serocellular crusts, orthokeratotic hyperkeratosis with gram-positive cocci and gram-negative, short bacilli; multifocal, moderate heterophilic, lymphoplasmacytic dermatitis with edema, necrosis and myriad, deep gram-negative short bacilli and fewer gram-positive cocci	Renal ductal trematodes	Bacterial dermatitis, arthritis, tenosynovitis and osteomyelitis with skin ulceration and crusting<br>Poor nutritional condition
**W20-456D**	Lameness<br>Lethargy<br>Swollen joint (legs/digits, wings)<br>Radiographs: Osteomyelitis, right carpus, right digit 3, left hock, left digit 3, left and right metatarsophalangeal joints<br>Euthanized in clinic	185.0	Carpus: moderate unilateral (right) swelling, discoloration (purple), and rigidity<br>Hock: mild bilateral swelling<br>Tarsometatarsophalangeal joints and digits: mild bilateral swelling	Skin scabs over right hock and plantar right and left 3rd digits	Not examined	Not examined	Not examined	Not examined	Presumed bacterial dermatitis, arthritis, tenosynovitis and osteomyelitis with skin scabbing<br>Poor nutritional condition
**W20-456E**	Lameness<br>Lethargy<br>Swollen joint (legs/digits, wings)<br>Radiographs: Osteomyelitis, left hock, left metatarsophalangeal joint, left digit 2, right digits 2 and 4<br>Euthanized in clinic	145.0	Tarsometatarsophalangeal joints: moderate bilateral swelling<br>Digits: mild to moderate bilateral swelling (right digits 2-4; left digits 3-4)<br>Hock: mild bilateral swelling	Skin scabs, bilateral over hocks and plantar feet<br>Ulcers, lateral left digits 3 and 4	Not examined	Not examined	Not examined	Not examined	Presumed bacterial dermatitis, arthritis, tenosynovitis and osteomyelitis with skin scabbing and ulceration<br>Poor nutritional condition
**W21-481A[2]**	Lameness<br>Lethargy<br>Swollen joints (legs/digits)<br>Euthanized in field	197.0	Noted in field but specific joints not reported (severe hock lesions noted by histopathology)	None reported	None reported	Hock: Severe, multifocal, necrotizing, heterophilic tenosynovitis, arthritis and myositis with gram-positive cocci	Multifocal, severe, subacute epidermal ulceration and crusting with myriad gram-positive cocci; multifocal, moderate to severe, subacute, heterophilic, histiocytic and lymphoplasmacytic dermatitis with dermal necrosis	Focally extensive, severe, pancreatic necrosis with fibrosis	Staphylococcal dermatitis, arthritis, tenosynovitis and osteomyelitis with skin ulceration and crusting<br>Emaciated

**W21-481B**[2]	Lameness<br>Lethargy<br>Swollen joints (legs/digits)<br>Euthanized in field	237.0	Noted in field but specific joints not reported (severe hock lesions noted by histopathology)	None reported	None reported	Hock: Mild to moderate, multifocal, necrotizing, heterophilic tenosynovitis, arthritis and myositis with chondrocyte degeneration	Not examined	Focally extensive, moderate, pancreatic necrosis with fibrosis<br>Renal trematodes	Staphylococcal dermatitis, arthritis and tenosynovitis<br>Poor nutritional condition
**W21-481C**	Lameness<br>Lethargy<br>Swollen joints (legs/digits)<br>Died in transit	180.4	Hock: Mild bilateral joint swelling	None reported	Multifocal renal pallor with nodules<br>Ventricular plasticosis	Not examined	Not examined	Focal, mild, acute, necroheterophilic nephritis with bacteria<br>Necroheterophilic, mild, multifocal, acute pneumonia	Staphylococcal arthritis (gross diagnosis)<br>Sepsis (*Staphylococcus aureus*)<br>Poor nutritional condition
**W21-481D**	Lameness<br>Lethargy<br>Swollen joints (legs/digits)<br>Died in transit	239.6	Hock: Severe unilateral (right) joint swelling	Skin ulcer with crusting, left hock	Ventricular plasticosis	Hock: Multifocal to coalescing, severe, subacute necroheterophilic tenosynovitis with gram-positive cocci	Not examined	Mild splenic heterophilia	Staphylococcal arthritis, tenosynovitis and osteomyelitis with skin ulceration and crusting<br>Poor nutritional condition
**W21-481E**[2]	None reported<br>Manner of death unknown	281.5	None reported	Interdigital webbing (skin) lacerations, right foot	None reported	Not examined	None	Spindle cell hepatocellular carcinoma	Hepatic spindle cell tumor<br>Poor nutritional condition
**W21-517B**[2]	Lameness<br>Alert<br>Swollen joints (legs/digits, wings)<br>Euthanized in clinic	254.0	Hock: moderate (left) to severe (right), bilateral swelling<br>Digits and plantar foot: moderate unilateral (right) swelling and reddening	Skin abrasion, right digit 3<br>Puncture and nodular reddening, interdigital foot webbing<br>Hemorrhage, medial right wing and pericloacal<br>Sandspur with hemorrhage and crusting over left pelvis	None reported	Hock and tarsometatarsophalanges: Multifocal, severe, subacute, heterophilic tenosynovitis and osteomyelitis with edema and gram-positive cocci	Multifocal (rare), mild epidermal ulceration with serocellular crusting; multifocal, mild heterophilic dermatitis with edema, necrosis and gram-positive cocci	Diffuse, mild to moderate hepatic cord atrophy	Staphylococcal dermatitis, arthritis, tenosynovitis, and osteomyelitis with skin ulceration, crusting and sandspurs<br>Poor nutritional condition

**W21-517C**[2]	Lameness<br>Lethargic<br>Swollen joints (legs/digits, wings)<br>Euthanized in clinic	254.0	Hock: moderate (right) to severe (left), bilateral with subcutaneous edema	Skin scabs, bilateral over feet<br>Skin abrasions and lacerations, right femur and plantar left digits<br>Reddening and nodular swelling caudoventral abdomen; reddening and multifocal abrasions skin ventral right wing; dried blood over ventral left elbow<br>Puncture wounds, ventral left metatarsal joint, digits 3 and 4 interdigital webbing, right femoral-tibiotarsal joint<br>Hemorrhage, right cranial coelom (thoracic region), ventral left elbow<br>Two sandspurs in feathers over pelvis	None reported	Hock, tarsometatarsophalanges, digits: Multifocal, severe, subacute to chronic, heterophilic, granulomatous tenosynovitis and osteomyelitis with hyperkeratosis, edema and gram-positive cocci	Multifocal, moderate, subacute, epidermal ulceration with serocellular crusts and hyperkeratosis; multifocal, moderate, heterophilic and granulomatous dermatitis with necrosis, fibrin, edema, and gram-positive cocci	None	Staphylococcal dermatitis, arthritis, tenosynovitis, and osteomyelitis with skin ulceration, crusting and sandspurs
**W21-673A**[2]	Lameness<br>Swollen joints (legs/digits)<br>Manner of death unknown	Unknown	Hock: severe bilateral swelling<br>Digits and plantar surface: mild (left) to moderate (right) bilateral swelling with discoloration (i.e., dark/purple)	Punctures and full thickness skin lacerations, bilaterally interdigital webbing; focal crust 3rd digit; multifocal nodular pale foci and reddening of webbing<br>Hemorrhage, skin over caudal coelom (medial to right leg)<br>Two embedded sandspur spikes left foot	Renal pallor with reticular pattern	Hock: Multifocal, mild, subacute to chronic, heterophilic and necrotizing tendinitis and periosteitis<br>Digits: Multifocal, moderate, subacute to chronic, heterophilic, granulomatous and necrotizing osteomyelitis, tenosynovitis with gram-positive cocci	Multifocal, moderate, heterophilic, granulomatous, necrotizing dermatitis with multifocal hyperkeratosis and gram-positive cocci	Multifocal, mild, acute, renal tubular degeneration with urate stasis	Staphylococcal dermatitis, arthritis, tenosynovitis, and osteomyelitis with skin punctures and sandspurs<br>Sepsis (*Staphylococcus aureus*)<br>Emaciation

**W21-673B**[2]	Lameness<br>Swollen joints (legs/digits)<br>Euthanized in field	Unknown	Hock: severe bilateral swelling<br>Digits and plantar foot: severe bilateral nodular swelling (left digit 3 and right digits 2-4) with reddening and crusting; right plantar ulcerated nodule	Ulcerated nodule, skin over right foot digit<br>Multifocal pale nodular foci dorsal webbing with crusting	None reported	Digits: Multifocal, severe, subacute to chronic, heterophilic, granulomatous and necrotizing osteomyelitis and tenosynovitis and gram-positive cocci	Multifocal, marked, heterophilic and granulomatous dermatitis with necrosis and gram-positive cocci	None	Staphylococcal dermatitis, arthritis, tenosynovitis, and osteomyelitis with skin ulceration and crusting<br>Sepsis (*S. aureus*)<br>Emaciation
**W21-673D**[2]	Lameness<br>Swollen joints (legs/digits)<br>Euthanized in clinic	187.0	Hock: Severe, bilateral swelling<br>Digits and foot plantar surface: severe bilateral nodular swelling, right digits 1, 3, 4; amputated digit 2 at distal phalanx and left foot digit 3	Punctures, multifocal in skin of interdigital webbing with crusting<br>Ulcerated nodule, skin over plantar right foot<br>Embedded sandspur spikes, foot webbing	Intestinal nematodes	Digits: Multifocal, severe, subacute to chronic, heterophilic, granulomatous and necrotizing osteomyelitis, tenosynovitis with gram-positive cocci	Multifocal, severe serocellular crusting with superficial gram-positive cocci and hyperkeratosis; necroheterophilic and granulomatous dermatitis with edema and gram-positive cocci	None	Staphylococcal dermatitis, arthritis, tenosynovitis, and osteomyelitis with skin punctures, crusting and sandspurs<br>Poor nutritional condition
**W21-673E**[2]	Lameness<br>Swollen joints (legs/digits)<br>Euthanized in field	143.0	Hock: moderate bilateral swelling<br>Digits: mild bilateral nodular swelling	Nodular swelling, bilateral in tarsal/metatarsal joints<br>Skin crusts, bilateral in dorsal and plantar interdigital webbing	None reported	Digits: Multifocal, severe, subacute to chronic, heterophilic, granulomatous and necrotizing osteomyelitis and tenosynovitis with gram-positive cocci<br>Hock: Focal, moderate epidermal ulceration with superficial gram-positive cocci and heterophilic dermatitis	Focal, epidermal ulceration with hyperkeratosis, serocellular crusts and gram-positive cocci; marked heterophilic and granulomatous dermatitis	None	Staphylococcal dermatitis, arthritis, tenosynovitis, and osteomyelitis with skin ulceration and crusting<br>West Nile virus infection<br>Poor nutritional condition
**W21-673G**[2]	Lameness<br>Swollen joints (legs/digits)<br>Euthanized in field	178.5	Hock: mild unilateral (left) joint swelling<br>Digits and plantar foot: bilateral mild (right) to moderate (left) nodular swelling with medial deviation of left foot digits with reddening	Skin crusting proximal to hock<br>Sandspurs, multifocally on ventral wings with hemorrhage; ≥ten additional sandspurs on body<br>Embedded sandspur spikes in foot webbing with reddening	None reported	Digits: Multifocal, severe, subacute to chronic, heterophilic, granulomatous and necrotizing osteomyelitis and tenosynovitis with gram-positive cocci	Multifocal, severe, heterophilic and granulomatous dermatitis with necrosis, gram-positive cocci and epidermal crusting	None	Staphylococcal dermatitis, arthritis, tenosynovitis, and osteomyelitis with skin crusting and sandspurs<br>Poor nutritional condition

**W21-673H**[2]	Lameness<br>Swollen joints (legs/digits)<br>Euthanized in field	Unknown	Hock: severe bilateral swelling with unilateral (right), deep open wound exposing the joint<br>Digits: severe bilateral swelling	Skin crusting, bilateral on plantar feet<br>Lacerations and hemorrhage over left femur with entangled sandspurs<br>Entangled sandspurs with hemorrhage, bilaterally on ventral wings	None reported	Digits: Multifocal, severe, subacute to chronic heterophilic, granulomatous and necrotizing osteomyelitis and tenosynovitis with gram-positive cocci	Multifocal, severe, subacute epidermal ulceration, necrosis and serocellular crusting; Multifocal, severe, subacute, heterophilic to granulomatous dermatitis with necrosis and gram-positive cocci	Mild, acute, focally extensive, heterophilic pneumonia	Staphylococcal dermatitis, arthritis, tenosynovitis, and osteomyelitis with skin ulceration, crusting and sandspurs<br>Staphylococcal pneumonia (presumed sepsis)<br>West Nile virus infection<br>Emaciation
**W21-673I**	Lameness<br>Swollen joints (legs/digits)<br>Manner of death unknown	229.0	Digits: severe, bilateral (left worse than right) with reddening	Skin scabbing and reddening, multifocally on plantar interdigital webbing and feet<br>Swollen webbing left digits 3 and 4	Fracture, left humerus near proximal epiphysis	Digits: Multifocal, moderate, subacute heterophilic, lymphoplasmacytic tenosynovitis with chondrocyte degeneration	Multifocal, severe, chronic, epidermal ulceration, serocellular crusting and gram-positive cocci; multifocal, moderate heterophilic, lymphocytic dermatitis	Mild intestinal (intraluminal) cestodes	Staphylococcal dermatitis, arthritis and tenosynovitis with skin ulceration and crusting<br>Poor nutritional condition
**W22-520A**	Swollen joints (legs/digits)<br>Died in field	278.6	Hock and stifle: mild, bilateral swelling with discoloration (i.e., purple) over stifle<br>Digits: moderate (right) to severe (left) bilateral swelling and discoloration (i.e., dark red to purple)	Skin ulcer over dorsal hock<br>Sandspurs embedded axillary region	Esophageal (intraluminal) nematode	Hock: Multifocal to coalescing, severe, subacute, heterophilic and granulomatous tenosynovitis, arthritis and myositis with gram-positive cocci<br>Digit: Multifocal to coalescing, severe, subacute, heterophilic and granulomatous tenosynovitis and arthritis with gram-positive cocci<br>Stifle: Focal, moderate, heterophilic and granulomatous periarthritis	Focal, severe, epidermal ulceration with gram-positive cocci; multifocal, moderate, heterophilic, histiocytic dermatitis with edema and fibroplasia	None	Staphylococcal dermatitis, arthritis and tenosynovitis with skin ulceration and sandspurs<br>Sepsis (*Staphylococcus aureus*)<br>Emaciation

**W22-520B**	Swollen joints (legs/digits)<br>Died in field	193.6	Hock: bilateral mild (left) to moderate (right) swelling<br>Digits: bilateral moderate (left digit 3) to severe (right digits 1-4) swelling with discoloration (i.e., purple)	Skin ulcers, multifocal on right foot<br>Sandspurs embedded, axillary region	Splenomegaly	Digit: Multifocal to coalescing, severe, subacute, heterophilic and granulomatous tenosynovitis with gram-positive cocci<br>Hock: Focal, mild, acute, heterophilic tenosynovitis<br>Stifle: Marked, necrotizing synovitis with chondrocyte degeneration; focally extensive, severe, chronic, granulomatous, periosteal myositis with femoral and tibiotarsal myeloid hyperplasia	Multifocal, marked epidermal ulceration, necrosis and hyperkeratosis;<br>Multifocal to coalescing, severe, subacute, granulomatous dermatitis with necrosis and gram-positive cocci	None	Staphylococcal dermatitis, arthritis and tenosynovitis with skin ulceration and sandspurs<br>Emaciation
**W22-520C**	Swollen joints<br>Died in field	161.1	Hock: severe bilateral swelling<br>Digits: mild (left digits 1-2) to moderate (right digit 2) bilateral swelling	Puncture, caudal right metatarsus<br>Sandspurs embedded axillary region	None reported	Hock: Multifocal, severe, subacute, heterophilic and granulomatous tenosynovitis and arthritis with gram-positive cocci<br>Digit: Diffuse, moderate, subacute, heterophilic tenosynovitis with focally extensive granulation tissue<br>Stifle: Diffuse, moderate, subacute, necroheterophilic synovitis	Multifocal to coalescing, subacute to chronic, necroheterophilic dermatitis with gram-positive cocci, fibroplasia and edema	Multifocal, moderate, acute, renal tubular necrosis<br>Multifocal, mild, acute, necrotizing pneumonia<br>Focal, mild, acute, myocardial necrosis	Staphylococcal dermatitis, arthritis and tenosynovitis with skin puncture and sandspurs<br>Sepsis (*Staphylococcus aureus*)<br>Emaciation

**W22-647**	None reported<br>Died in clinic	265.0	None reported	None reported	Renal pallor with reticular pattern	Not examined	None	Multifocal to coalescing, severe, subacute, necrotizing enteritis with gram-negative bacilli<br>Multifocal, severe, subacute, renal tubular necrosis with heterogranulomatous nephritis and gram-negative bacilli<br>Multifocal, mild, subacute, hepatic necrosis with granulomatous hepatitis and gram-negative bacilli<br>Multifocal, mild, subacute, heterophilic cloacal bursitis with gram-negative bacilli<br>Multifocal, mild, subacute, heterophilic and granulomatous pneumonia; diffuse, moderate, splenic lymphoid depletion<br>Bone marrow serous atrophy of fat with gram-negative bacilli	Disseminated bacterial infection with gram-negative bacilli<br>Poor nutritional condition
**Distant colonies unaffected by staphylococcal arthritis (northwest coast)**									
**W21-673C**[2]	None reported<br>Died in field	125.0	None reported	None reported	None	None	None	Focal pulmonary parabronchial bacterial cocci	Emaciation
**W21-673F**	None reported<br>Died in field	Unknown	None	Mild bilateral dermal swelling with skin crusting over hock region and distal phalanges and plantar feet with nodular swelling and reddening in the latter	None	None	Moderate serocellular crusting, gram-positive cocci in crust and dermis with acute dermal necrosis	Renal ductal trematodes	Dermal necrosis with skin crusting<br>Emaciation

**W22-410A**	Lethargic<br>Recumbent (unable to rise)<br>Died in field	198.5	None	None reported	Multifocal to coalescing, pale hepatic foci<br>Mild myocardial petechiae<br>Few proventricular and ventricular nematodes	None	None	Multifocal to coalescing, severe, subacute hepatic necrosis with hemorrhage<br>Multifocal, acute, moderate, ulcerative typhlitis with mixed gram-positive and negative bacteria<br>Renal tubular luminal mineralized concretions<br>Small intestinal trematodes	Emaciation<br>Hepatitis (suspect bacterial)
**W22-410B**	Lethargic<br>Died in field	60.1	None	Skin scabs, left (oral) commissure<br>Skin abrasions, right flank	Oral and esophageal trematodes	None	None	Esophageal intraluminal trematode	Emaciation
**W22-486**	Lethargic<br>Neck contusions<br>Died in field	122.6	None	None reported	Focal ecchymosis, skin over lateral neck	None	None	Small intestinal and cloacal luminal trematodes	Emaciation
**W22-645**[2]	Lethargic<br>Died in clinic	124.0	None reported	Mild skin ulcer, distal right limb	None	None	None	Renal intratubular mineral	Emaciation<br>Skin ulceration
**W23-482A**	Lethargic<br>Died in transit	135.4	None	Puncture with sandspurs over neck and leg joints	None	None	Focal, moderate epidermal ulceration with serocellular crusting and heterophilic dermatitis	Renal intratubular urate stasis	Chronic dermatitis (neck/legs/digits) with skin puncture/penetrating sandspur wounds<br>Emaciation (starvation)
**W23-482B**	Lethargic<br>Died in clinic	67.3	None	Hock and digits: mild unilateral dermal swelling over right hock and digits	None	None	Multifocal, moderate ulceration with serocellular crusting with focal, mild heterophilic dermatitis (with no visible bacteria by Gram stain)	Cloacal bursal lymphoid depletion<br>Multifocal, marked, heterophilic, granulomatous nephritis (no visible bacteria by Gram stain) with intratubular urates<br>Focal (cervical) perivertebral hemorrhage	Chronic dermatitis (neck/legs/digits) with skin ulceration<br>Emaciation (starvation)

**W23-482C**	Lethargic<br>Died in transit	49.8	None	None reported	None	None	Focal, acute ulceration with moderate, multifocal, heterophilic dermatitis	Moderate, multifocal, acute, necrotizing, heterophilic cloacal bursal folliculitis with extracellular gram-positive cocci and bursal lymphoid depletion<br>Renal intratubular urate stasis	Chronic dermatitis (neck/legs/digits) with skin ulceration<br>Emaciation (starvation)
**W23-482D**	Died in field	81.0	None	Embedded sandspur, skin over plantar right foot<br>Hemorrhage, subcutaneous proximal neck	Multifocal, pinpoint, renal pallor<br>Focally extensive subcutaneous hemorrhage over neck	None	Focal, severe epidermal ulceration and serocellular crusting with gram-positive cocci and fibrinoheterophilic dermatitis with hemorrhage and edema	Cloacal bursal lymphoid depletion<br>Renal tubular urate stasis<br>Hepatocellular atrophy	Chronic dermatitis (neck/legs/digits) with skin puncture/penetrating sandspur wounds<br>Emaciation (starvation)
**W23-482E**	Lethargic<br>Died in clinic	114.1	None	None reported	None	Not examined	Not examined	Not examined	Emaciation (starvation)
**W23-482F**	Lethargic<br>Died in transit	232.4	None	Puncture with hemorrhage over plantar left hock	None	Not examined	Not examined	Not examined	Emaciation (starvation)

[1] All skimmers were in poor to emaciated nutritional condition with variable skeletal muscle atrophy, absent adipose stores, and bone marrow serous atrophy of or severe depletion of fat; most also had scant gastrointestinal contents and urate stasis and/or mineral deposits in renal tubules, consistent with dehydration (not included in gross or histopathologic findings).

[2] Necropsy performed at Florida Fish and Wildlife Conservation Commission facilities; all others necropsied at the Southeastern Cooperative Wildlife Disease Study.

[3] Joints: Digits-interphalangeal; Hock - tibiotarsal-metatarsal; Stifle - femoral-tibiotarsal; Carpus - ulnar-radial-carpometacarpal; Elbow - femoral-ulnar-radial; Tarsometatarsal-phalangeal.

[4] Parasitic infections are considered incidental based on parasite local and load but may have been facilitated by immune challenge or immunosuppression.

Supplemental Table 2: Antimicrobial susceptibility pattern for Staphylococcus aureus isolates from juvenile black skimmers with staphylococcal polyarthritis in from nest colonies along southwest Florida from 2020-2023.[1]

	W20-456A joint		W20-456B joint		W21-517B joint		W21-673A lung		W21-673B foot joint		W22-520A toe joint		W22-520C kidney	
	Int[3]	MIC	Int	MIC	Int	MIC	Int	MIC	Int	MIC	Int	MIC	Int	MIC
Amikacin	S	≤2	S	≤2	S	≤2	S	≤2	S	≤2	S	≤2	S	≤2
Amoxicillin/clavulanic acid	S	≤2	S	≤2	S	≤2	S	≤2	S	≤2	S	≤2	S	≤2
Benzylpenicillin	S	0.12	S	0.12	S	0.06	S	0.06	S	0.06	S	0.06	S	0.06
Beta-Lactamase	—	Neg	—	Neg	—	Neg	—	Neg	—	Neg	—	Neg	—	Neg
Cefovecin (3rd gen.)	S	1	S	1	S	1	S	1	S	1	S	1	S	1
Cefpodoxime (3rd gen.)	S	2	S	2	S	2	I	4	S	2	S	2	S	2
Chloramphenicol	S	8	S	8	S	8	S	8	S	8	S	8	S	8
Clindamycin	S	0.25	S	0.25	S	0.25	S	0.25	S	0.25	S	0.25	S	0.25
Doxycycline[2]	S	≤0.5	S	≤0.5	S	≤0.5	S	≤0.5	S	≤0.5	S	≤0.5	S	≤0.5
Enrofloxacin	S	≤0.5	S	≤0.5	S	≤0.5	S	≤0.5	S	≤0.5	S	≤0.5	S	≤0.5
Erythromycin	S	≤0.25	S	≤0.25	S	≤0.25	S	≤0.25	S	≤0.25	S	≤0.25	S	≤0.25
Florfenicol	S	≤4	S	≤4	S	≤4	S	≤4	S	≤4	S	≤4	S	≤4
Gentamicin	S	≤0.5	S	≤0.5	S	≤0.5	S	≤0.5	S	≤0.5	S	≤0.5	S	≤0.5
ICR	—	Neg	—	Neg	—	Neg	—	Neg	—	Neg	—	Neg	—	Neg
Marbofloxacin	S	≤0.5	S	≤0.5	S	≤0.5	S	≤0.5	S	≤0.5	S	≤0.5	S	≤0.5
Minocycline	S	≤0.5	S	≤0.5	S	≤0.5	S	≤0.5	S	≤0.5	S	≤0.5	S	≤0.5
Nitrofurantoin	S	≤16	S	≤16	S	≤16	S	≤16	S	≤16	S	≤16	S	≤16
Oxacillin	S	≤0.25	S	≤0.25	S	≤0.25	S	≤0.25	S	≤0.25	S	≤0.25	S	≤0.25
Pradofloxacin	S	≤0.12	S	≤0.12	S	≤0.12	S	≤0.12	S	≤0.12	S	≤0.12	S	≤0.12
Trimethoprim/Sulfa	S	≤10	S	≤10	S	≤10	S	≤10	S	≤10	S	≤10	S	≤10

[1] This in vitro test may not accurately reflect clinical outcome.

[2] According to the Athens Veterinary Diagnostic Laboratory, the panel's calling range for doxycycline with Enterobacteriales and *Staphylococci* is based on human criteria (i.e., ≤4: Susceptible, ≥16: Resistant).

[3] Int - interpretation; MIC - minimum inhibitory concentrations; S - susceptible; I - intermediate; ICR - inducible clindamycin resistance.

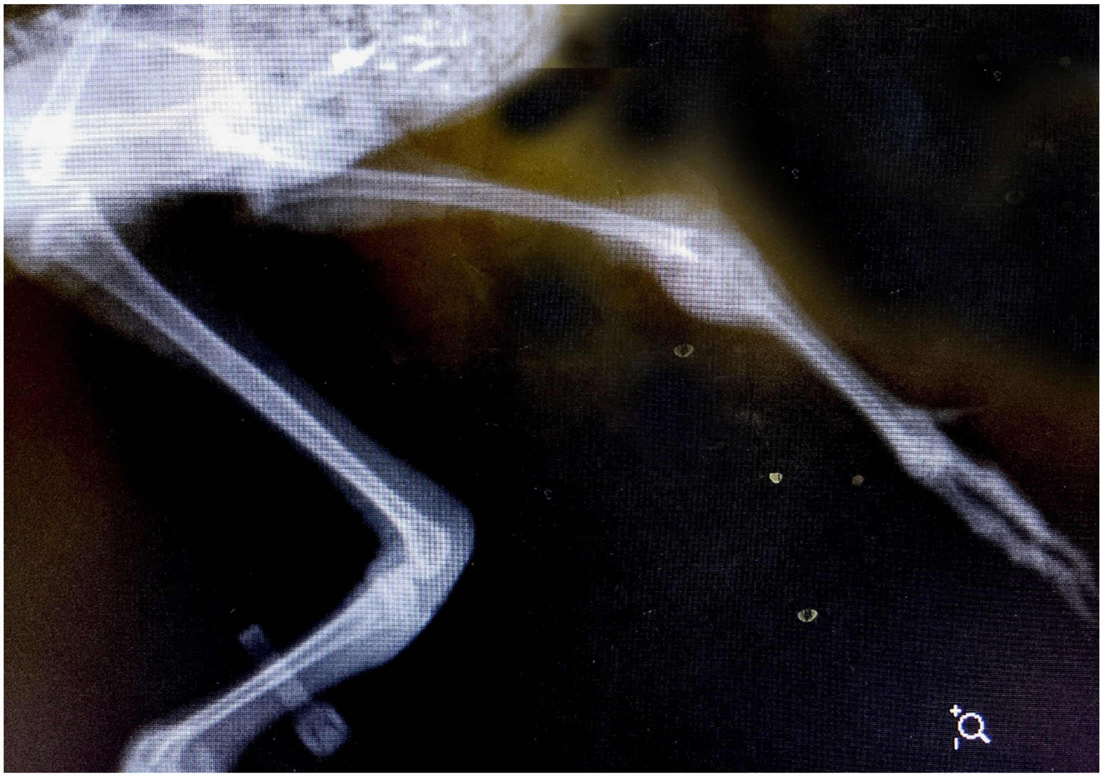

Figure S1. Radiographs revealing marked swelling of soft tissue region surrounding the tibiotarsal-tarsometatarsal (hock) joint in a black skimmer with staphylococcal arthritis.

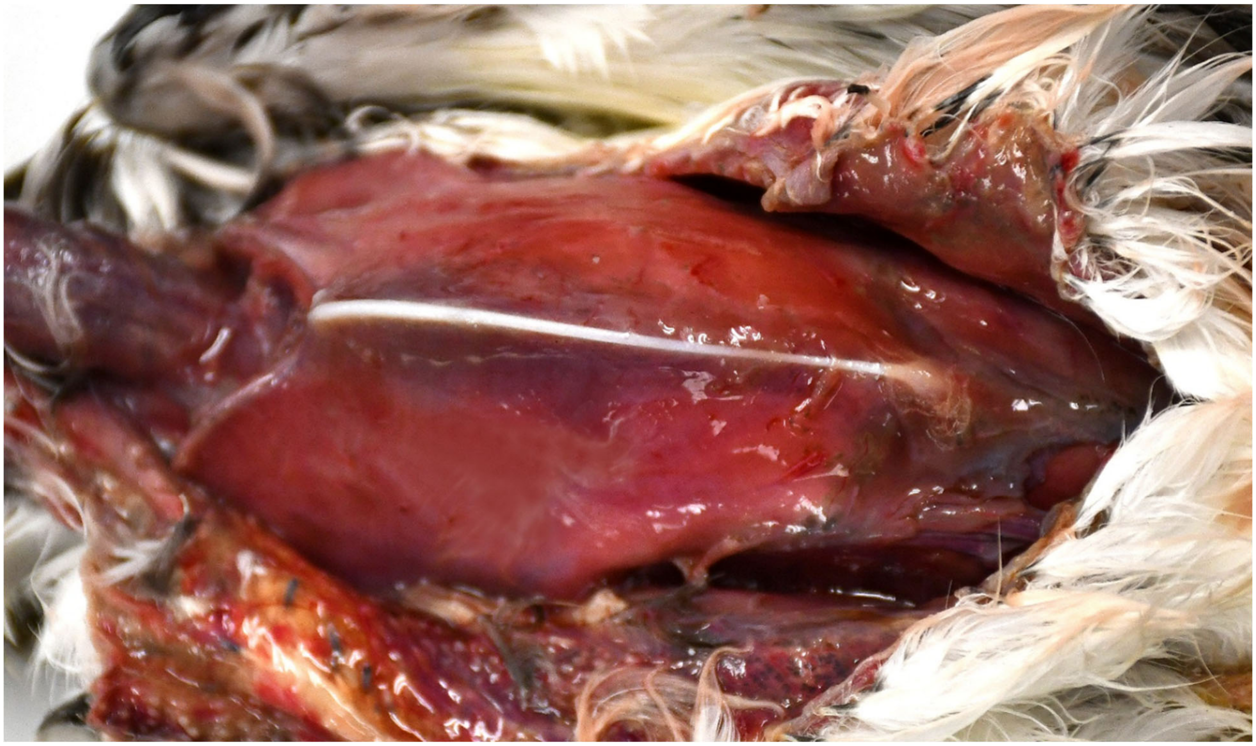

Figure S2. Poor nutritional condition with absent adipose stores and markedly atrophic skeletal musculature in a black skimmer with staphylococcal arthritis.

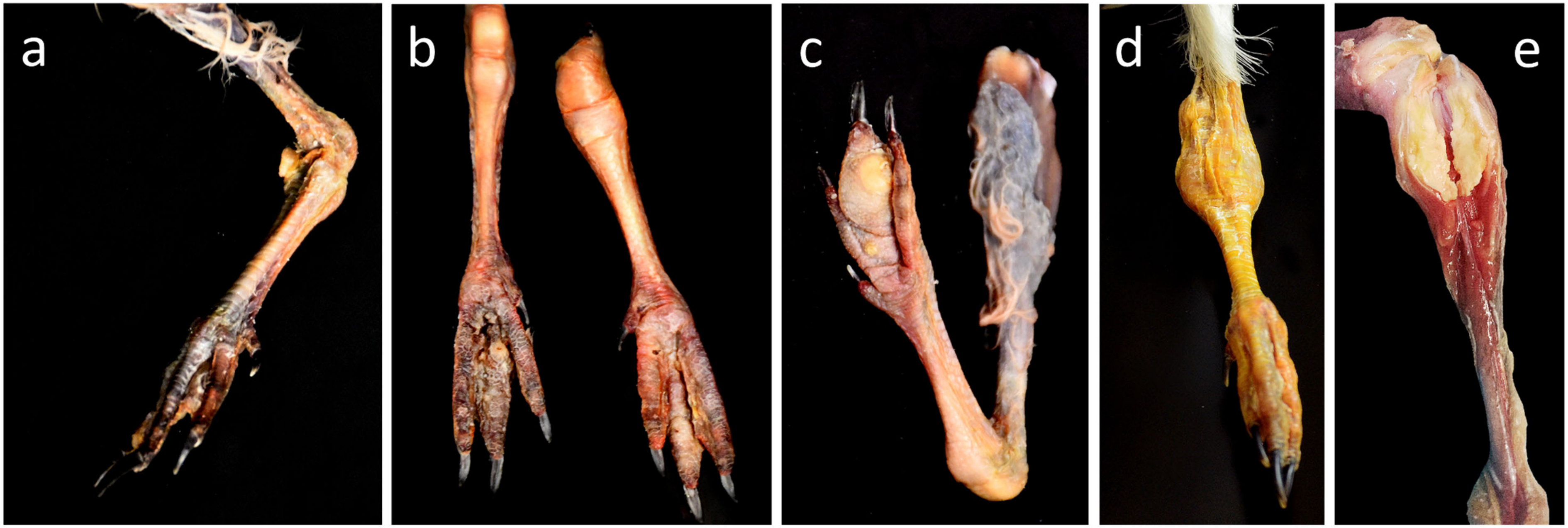

Figure S3. Black skimmers with staphylococcal arthritis suspected to be associated with sandspurs often exhibited skin ulceration and necrosis (evident as darkening and sloughing) (a, b), marked swelling of one or more joints (b, c, d, e) with pale, yellow, soft to firm material (inflammation; c, e). Joint disease most often involved interphalangeal (digits, a, b, c) and tibiotarsal-metatarsal (hock, b, c, d, e) joints. Figure 3e reveals abundant inflammatory exudate in a transected joint.
